# Core outcome measures for clinical effectiveness trials of nutritional and metabolic interventions in critical illness: an international modified Delphi consensus study evaluation (CONCISE)

**DOI:** 10.1186/s13054-022-04113-x

**Published:** 2022-08-06

**Authors:** T. W. Davies, R. J. J. van Gassel, M. van de Poll, J. Gunst, M. P. Casaer, K. B. Christopher, J. C. Preiser, A. Hill, K. Gundogan, A. Reintam-Blaser, A. F. Rousseau, C. Hodgson, D. M. Needham, M. Castro, S. Schaller, T. McClelland, J. J. Pilkington, C. M. Sevin, P. E. Wischmeyer, Z. Y. Lee, D. Govil, A. Li, L. Chapple, L. Denehy, J. C. Montejo-González, B. Taylor, D. E. Bear, R. Pearse, A. McNelly, J. Prowle, Z. A. Puthucheary

**Affiliations:** 1 William Harvey Research Institute, Barts and The London School of Medicine and Dentistry, Queen Mary University of London, London, UK; 2 Critical Care and Perioperative Medicine Research Group, Adult Critical Care Unit, Royal London Hospital, London, E1 1BB UK; 3 Department of Intensive Care Medicine, School of Nutrition and Translational Research in Metabolism (NUTRIM), Maastricht University Medical Centre+, Maastricht, The Netherlands; 4 Department of Surgery, School of Nutrition and Translational Research in Metabolism (NUTRIM), Maastricht University Medical Centre+, Maastricht, The Netherlands; 5 Clinical Department and Laboratory of Intensive Care Medicine, Department of Cellular and Molecular Medicine, KU Leuven, Herestraat 49, 3000 Leuven, Belgium; 6 Division of Renal Medicine, Channing Division of Network Medicine, Brigham and Women’s Hospital, Boston, USA; 7 Medical Direction, Erasme University Hospital, Universite Libre de Bruxelles, Brussels, Belgium; 8 Departments of Intensive Care and Anesthesiology, University Hospital RWTH Aachen University, 52074 Aachen, Germany; 9 Division of Intensive Care Medicine, Department of Internal Medicine, Erciyes University School of Medicine, Kayseri, Turkey; 10 Department of Anaesthesiology and Intensive Care, University of Tartu, Tartu, Estonia; 11 Department of Intensive Care Medicine, Lucerne Cantonal Hospital, Lucerne, Switzerland; 12 Department of Intensive Care, University Hospital of Liège, Liege, Belgium; 13 Australian and New Zealand Intensive Care Research Centre, School of Public Health and Preventive Medicine, Monash University, 3/553 St Kilda Rd, Melbourne, VIC 3004 Australia; 14 Department of Intensive Care and Hyperbaric Medicine, The Alfred, Melbourne, VIC Australia; 15 Outcomes After Critical Illness and Surgery (OACIS) Research Group, Johns Hopkins University, Baltimore, MD USA; 16 Pulmonary and Critical Care Medicine, Department of Medicine, and Department of Physical Medicine and Rehabilitation, Johns Hopkins University School of Medicine, Baltimore, MD USA; 17 Clinical Nutrition, Hospital Israelita Albert Einstein, Sao Paulo, Brazil; 18 Department of Anesthesiology and Operative Intensive Care Medicine (CVK, CCM), Charité - Universitätsmedizin Berlin, Corporate Member of Freie Universität Berlin, Humboldt-Universität Zu Berlin, Berlin Institute of Health, Berlin, Germany; 19 School of Medicine, Klinikum Rechts Der Isar, Department of Anesthesiology and Intensive Care, Technical University of Munich, Munich, Germany; 20 Centre for Bioscience, Manchester Metropolitan University, John Dalton Building, Chester Street, Manchester, UK; 21 Department of Medicine, Division of Allergy, Pulmonary, and Critical Care Medicine, Vanderbilt University Medical Center, Nashville, TN USA; 22 Department of Anesthesiology, Duke University School of Medicine, DUMC, Box 3094 Mail # 41, 2301 Erwin Road, Durham, NC 5692 HAFS27710 USA; 23 Department of Anesthesiology, University of Malaya, Kuala Lumpur, Malaysia; 24 Institute of Critical Care and Anesthesia, Medanta: The Medicty, Gurugram, Haryana India; 25 Division of Respiratory and Critical Care Medicine, Department of Medicine, National University Hospital, National University Health System, Singapore, Singapore; 26 Department of Intensive Care Medicine, Woodlands Health, Singapore, Singapore; 27 Adelaide Medical School, Faculty of Health and Medical Sciences, The University of Adelaide, Adelaide, SA Australia; 28 The University of Melbourne, School of Health Sciences, Melbourne, Australia; 29 Department of Allied Health, Peter McCallum Cancer Centre, Melbourne, Australia; 30 Department of Intensive Care Medicine, Hospital Universitario 12 de Octubre, Madrid, Spain; 31 Department of Research for Patient Care Services, Barnes-Jewish Hospital, St. Louis, MO USA; 32 Department of Critical Care and Department of Nutrition and Dietetics, Guy´S and St Thomas’ NHS Foundation Trust, London, UK

**Keywords:** Metabolism, Nutrition, Core outcome set, Critical illness, Delphi

## Abstract

**Background:**

Clinical research on nutritional and metabolic interventions in critically ill patients is heterogenous regarding time points, outcomes and measurement instruments used, impeding intervention development and data syntheses, and ultimately worsening clinical outcomes. We aimed to identify and develop a set of core outcome domains and associated measurement instruments to include in all research in critically ill patients.

**Methods:**

An updated systematic review informed a two-stage modified Delphi consensus process (domains followed by instruments). Measurement instruments for domains considered ‘essential’ were taken through the second stage of the Delphi and a subsequent consensus meeting.

**Results:**

In total, 213 participants (41 patients/caregivers, 50 clinical researchers and 122 healthcare professionals) from 24 countries contributed. Consensus was reached on time points (30 and 90 days post-randomisation). Three domains were considered ‘essential’ at 30 days (survival, physical function and Infection) and five at 90 days (survival, physical function, activities of daily living, nutritional status and muscle/nerve function). Core ‘essential’ measurement instruments reached consensus for survival and activities of daily living, and ‘recommended’ measurement instruments for physical function, nutritional status and muscle/nerve function. No consensus was reached for a measurement instrument for Infection. Four further domains met criteria for ‘recommended,’ but not ‘essential,’ to measure at 30 days post-randomisation (organ dysfunction, muscle/nerve function, nutritional status and wound healing) and three at 90 days (frailty, body composition and organ dysfunction).

**Conclusion:**

The CONCISE core outcome set is an internationally agreed minimum set of outcomes for use at 30 and 90 days post-randomisation, in nutritional and metabolic clinical research in critically ill adults.

**Supplementary Information:**

The online version contains supplementary material available at 10.1186/s13054-022-04113-x.

## Background

As mortality continues to decrease from critical illness, patients, clinicians and public sector organisations are increasingly aware of the consequences of surviving critical illness. Severe, prolonged functional disabilities are common and can persist for up to five years [1]. Physical and mental health impairments result in adverse socioeconomic consequences for patients and carers, recognised as a growing public health issue [2].

Functional impairments are therefore appropriate, necessary and urgent outcomes for critical care research to target, broadening the list of patient-centred outcome measures for randomised controlled trials. Outcomes assessing physical function are likely to be amenable to metabolic and nutritional interventions. Muscle wasting occurs rapidly in critical illness and is the result of decreased protein synthesis and bioenergetic failure, and intramuscular inflammation [3, 4]. Once this has occurred, recovery of physical function is difficult, with high-quality trials of physical rehabilitation unable to consistently demonstrate improvements in patient outcomes [5].

Nutritional and metabolic interventions may increase muscle protein synthesis, lessen bioenergetics failure and decrease inflammation in these patients, improving outcomes [6]. However, measuring physical functional outcomes is not standard practice in critical illness trials. A recent systematic review highlighted the lack of physical functional data and variation in outcomes collected, limiting comparisons between trials, future systematic reviews and meta-analyses [7, 8].

Therefore, an international group of patients, clinicians and researchers were convened to establish a consensus on the minimum Core Outcome Set (COS) for the evaluation of metabolic and nutritional interventions in clinical research involving critically ill adult patients.

## Methods

The modified Delphi consensus methodology is well described, used extensively in COS-related projects and uses expert opinion to address questions when empirical data either cannot answer or do not exist in appropriate form [9]. Briefly this involves at least two rounds of participants voting on recommendations related to a study question. Voting is informed by results of preceding rounds and performed anonymously to prevent external influence [10]. These results are reported in keeping with the COS-STAR Statement, and the project was registered with the COMET initiative (https://www.comet-initiative.org/Studies/Details/1838) [11]. The Queen Mary Ethics of Research Committee approved the study protocol (QMREC20.241).

### Update of systematic review

The most recent relevant systematic review covered the period January 2000 to August 2018 [7]. We updated this (August 2018 to March 2021), following the Preferred Items for Systematic Reviews and Meta-Analyses (PRISMA) reporting guidelines and prospectively registered the review on PROSPERO (CRD42021242457). Full details are available in Additional file 1: Table S1 and Figure S1.

### Steering committee

An international multidisciplinary steering committee was convened to guide the research design, recruitment and development of the core outcome set. The committee included 27 members from Europe, North America, South America, Asia and Australia (Additional file 1: Table S2).

### Generation of preliminary list of outcome domains and measurement instruments

Outcome domains and measurement instruments were extracted from both systematic reviews (Additional file 1: Table S3). A large number of COS have been developed, or are in production, for critically ill patients [12]. Relevant domains were extracted, mapped to a standard taxonomy for COS development and presented to the steering committee, in addition to the current definition of post-intensive care syndrome [13, 14].

It was recognised that a clear interaction exists between the outcomes and the time point at which these are measured. Equally, the literature demonstrates heterogeneity of such time points. Time points extracted from the systematic review were put to a vote at the initial steering committee meeting, in combination with other relevant time points arising during discussion. Criteria for consensus for inclusion in the Delphi process was > 70% of participants voting in favour of inclusion.

### Participants

A large Delphi panel was convened to establish the COS domains and associated measurement instruments. The panel consisted of representatives from the three stakeholder groups: patients who have survived critical illness or their caregivers (family or carers), clinicians who care for critically ill patients with an interest in metabolic and nutritional interventions and clinical researchers who might apply the COS (Additional file 1: Table S4). To ensure appropriate representation, the protocol was presented to the relevant sections of the European Society of Intensive Care Medicine, American Society of Parenteral and Enteral Nutrition, the Indian Society of Critical Care Medicine, the Brazilian Society of Parenteral and Enteral Nutrition and the United Kingdom Intensive Care Society. Clinicians and researchers in the field of physical functional outcomes research were additionally recruited from the United States, Canada, Australia, Singapore and Malaysia. All participants who volunteered through this process were asked to recruit patient representatives through their relevant institutions and support charities, and to use their local networks to identify further relevant clinical and academic participants for screening.

### Consensus process

All survey rounds were delivered electronically using DelphiManager software (COMET Initiative, University of Liverpool, UK). Consensus was reached via a two-stage process, with each stage containing two to three scoring rounds and a steering committee or consensus meeting, similar to previous studies [15]. In stage 1, participants scored each outcome domain according to the Grading of Recommendations Assessment, Development and Evaluation (GRADE) scale ranging from 1 to 9 in terms of importance for inclusion (1–3, not important for inclusion; 4–6, important but not critical; 7–9, critical to include). Criteria for consensus for inclusion of a domain was a ‘critical-to-include’ rating of 7–9 in > 70% of all responses and ≤ 15% of all responses rating the domain or measurement instrument as ‘not important’ (i.e., score ≤ 3). In stage 2, participants scored each measurement instrument according to the above GRADE scale. Criteria for ‘essential’ inclusion was a ‘critical-to-include’ rating of 7–9 in > 70% of all responses and ≤ 15% of all responses rating the domain or measurement instrument as ‘not important’ (i.e., score ≤ 3). Criteria for ‘recommended’ inclusion was a ‘critical-to-include’ rating of 7–9 in > 60% of all responses and ≤ 15% of all responses rating the domain or measurement instrument as ‘not important’ (i.e., score ≤ 3). Following the Delphi process, the measurement instruments that reached consensus criteria for inclusion were discussed at the final consensus meeting. Consensus meeting participants voted on the inclusion of these in the final COS. Criteria for consensus for inclusion in the final COS was > 70% of participants at the consensus meeting voting in favour of inclusion.

### Stage 1 for core outcome domains

*Round 1* Domains extracted from data sources were presented to the steering committee, and these populated the initial Delphi round. The order of domains was randomised. Participants were asked to rate each of the preliminary domains without consideration of ‘how’ that domain will be assessed. Participants were able to provide additional comments or suggest additional domains for consideration. All additional domains suggested were reviewed by the project team, ensuring they represented a new contribution and were provided as new domains for voting in round 2.

*Round 2* Participants received feedback on the distribution of scores and the average score of each domain from each of the three stakeholder groups, along with their own score and were asked to re-evaluate domains, including any new domains that were suggested in round 1.

*Round 3* If > 70% of responses from at least one stakeholder group rated > 7 for a newly suggested domain during round 2, participants were given feedback on the distribution of scores and the average score of each domain from each of the three stakeholder groups, along with their own score and were asked to re-evaluate the newly suggested domains.

*Steering committee consensus meeting* The results of stage 1 were reviewed by the steering committee via online conference to ratify findings. If problems were raised, views from all participants were discussed and considered. If any changes to methodology were considered necessary, then additional voting was required. Criteria for consensus on the proposed change was > 70% of participants voting in favour.

### Stage 2 for outcome measurement instruments

Outcome measurement instruments extracted from the systematic review were mapped to the core domains reaching consensus in stage 1. These were presented to the steering committee for suggestion of additional instruments and final agreement, before populating the initial Delphi round. A similar two-round consensus process was used for the measurement instruments as outlined above. Instrument cards were provided to each participant containing a description of the measurement instrument and important information relating to its use (example instrument card can be found at: https://www.improvelto.com/instruments).

*Consensus meeting* All study participants were invited to an online meeting where the results of the Delphi process and psychometric data on measurement instruments that reached consensus were presented (Additional file 1: Table S5). Measurement instruments which reached consensus in the Delphi for ‘essential’ inclusion were discussed and a final decision on inclusion was reached by anonymous voting. Consensus for inclusion was > 70% of participants voting in favour. If < 70% of participants voted in favour of ‘essential’ inclusion, then an additional vote took place to consider the measurement instrument for ‘recommended’ inclusion. Consensus for inclusion was again > 70% of participants voting in favour. Measurement instruments which reached consensus in the Delphi for ‘recommended’ inclusion were discussed and a final decision on inclusion was reached by anonymous voting. Consensus for inclusion was > 70% of participants voting in favour. If < 70% of participants voted in favour of ‘recommended’ inclusion, then the measurement instrument was excluded from the final COS.

### Data analysis

Survey responses were summarised with descriptive statistics. In each round, data were excluded if the survey was not completed in full. The responses from different stakeholder groups were compared using two-tailed Mann–Whitney U or Student’s *T*-test, as appropriate. No mathematical correction was made for multiple comparisons.

## Results

### Systematic review

**Fig. 1:**
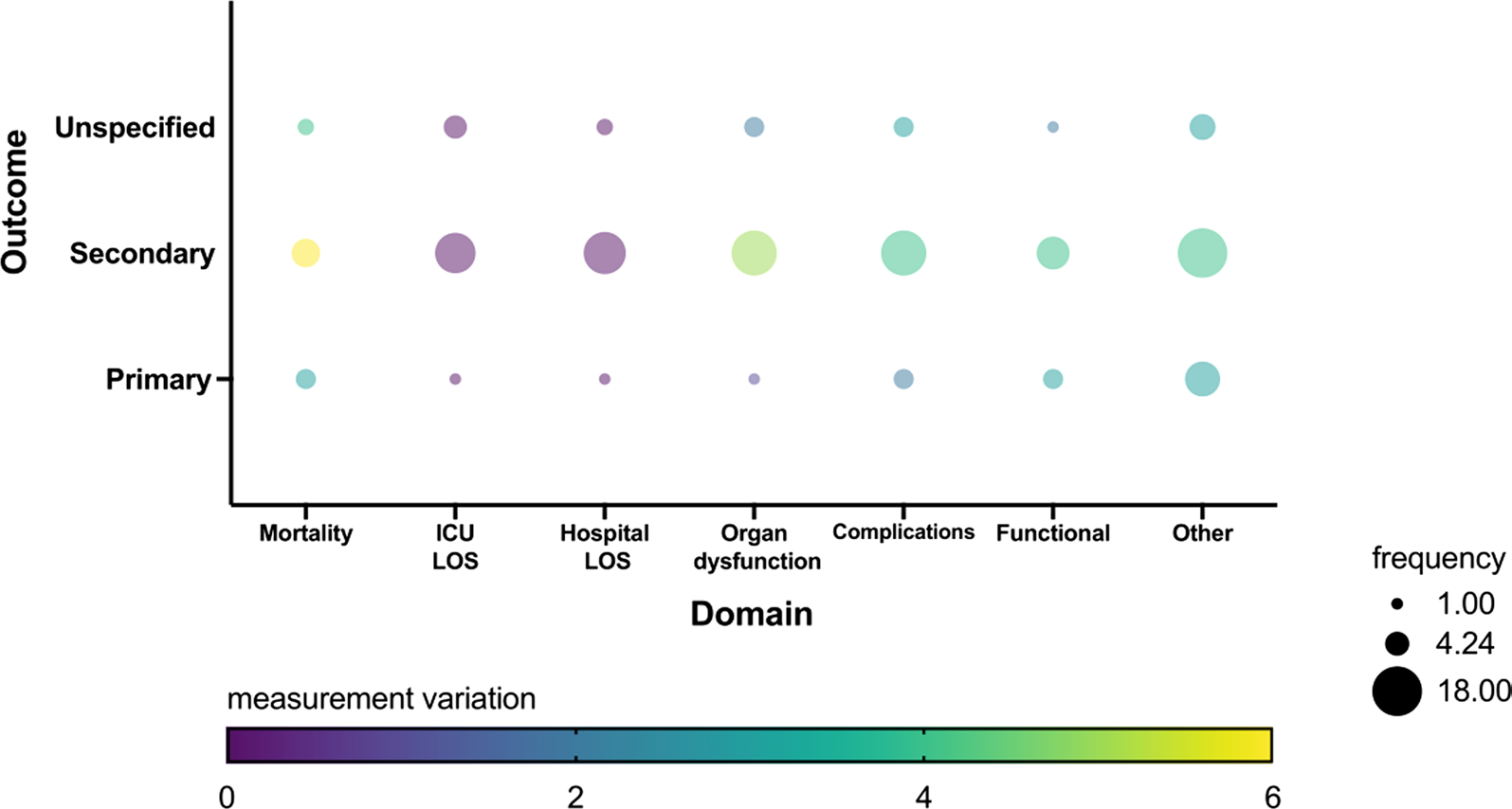
Graphic representation of outcomes identified in the updated systematic review. Bubble size denotes the frequency of outcome, bubble colour the variation in the definition of the measurement tool. Greater detail is available in Additional file 1

Twenty-five new trials were identified, covering nutritional strategies, composition and supplementation with varying measurement properties and time points (Fig. 1, Additional file 1: Tables S3, S6 and S7). Further details on the assessment for risk of bias are available in Additional file 1: Figures S2 and S3.

### Time points for COS measurements

Consensus was reached at the initial steering committee meeting (*n* = 11) on the use of fixed time points from randomisation (91%; 10/11). While a maximum of three points were discussed, consensus was reached only for 30 days (82%; 9/11) and 90 days (81%; 10/11) post-randomisation. No consensus was reached for a time point between 7 and 10 days (18%; 2/11) or for any other time point (intensive care unit (ICU) or hospital discharge). There was 100% agreement that for longer-term follow-up the previously published COS for acute respiratory failure survivors should be used [15].

### Delphi panel participants

**Fig. 2:**
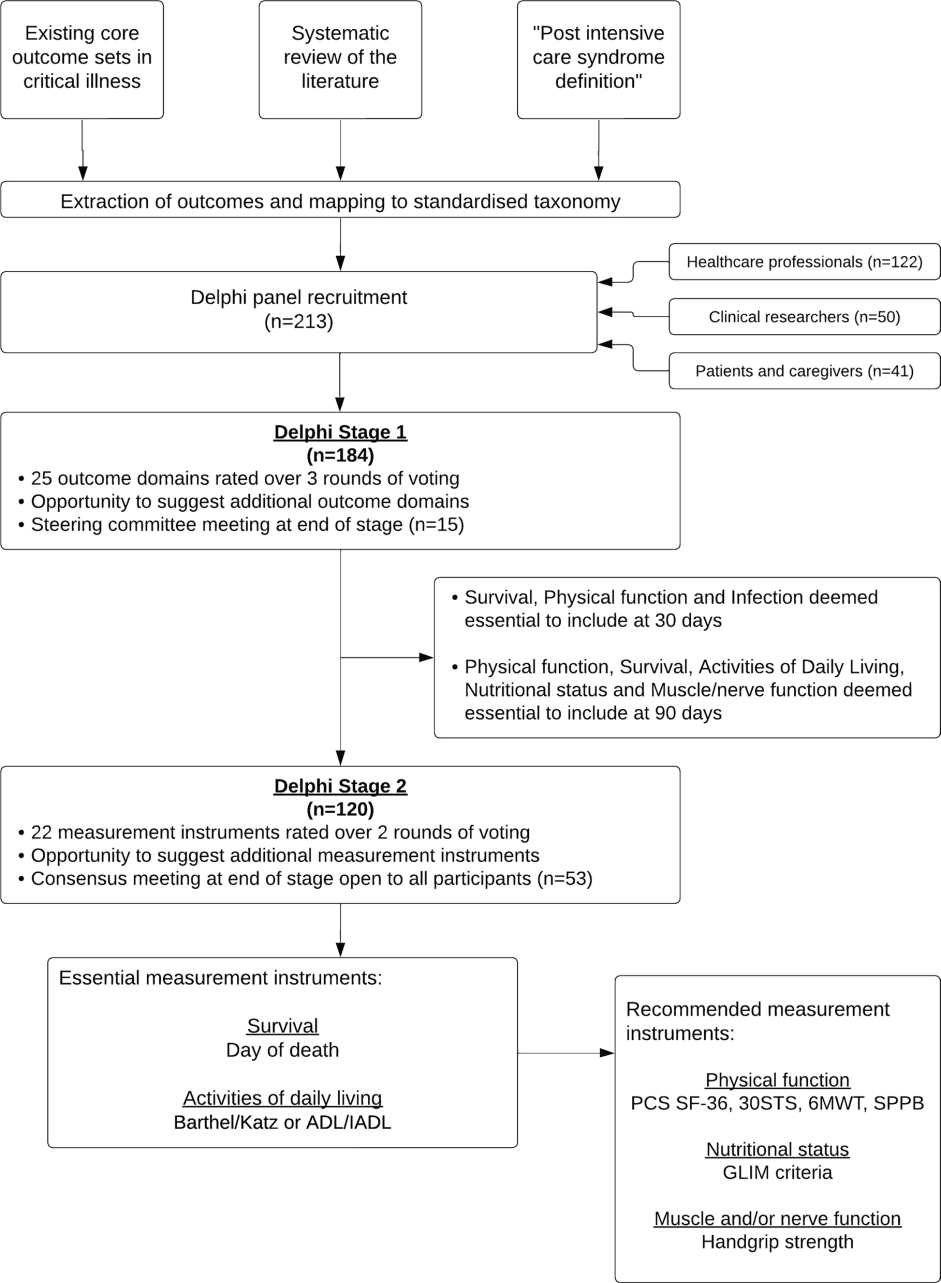
Modified Delphi Process Flow Diagram. 30STS = 30 s sit-to-stand; 6MWT = 6-min walk test; ADL = Activities of daily living; GLIM = Global leadership on malnutrition; IADL = Instrumental activities of daily living; PCS SF-36 = Physical component score of the short form 36; SPPB = Short physical performance battery

The international panel consisted of 213 participants from 24 countries (34 patients and 7 caregivers, 50 clinical researchers and 122 healthcare professionals). In each round, data were excluded where the survey was incomplete. After exclusion of incomplete data, the final number of included participants was 184 in stage 1 and 120 in stage 2 as shown in Fig. 2. The final consensus meeting included 53 participants (10 patients and caregivers, 23 clinical researchers and 20 healthcare professionals). Full details of participants and retention over the rounds are shown in Fig. 2, Additional file 1: Tables S4, S5 and Figure S4.

### COS domains

Fifteen domains at two time points (30 and 90 days from randomisation) were entered into the first round of stage 1 of the Delphi and 100% retained into the second round. Ninety-nine additional domains were suggested during the round, and after exclusion of duplicates, measurement instruments and existing domains, 10 additional domains were added. After round 2, a focused third round was needed to ensure that these 10 additional outcome domains had two rounds of voting if they reached consensus threshold in round 2 (Additional file 1: Tables S8 and S9). The results were discussed at the steering committee meeting following stage 1 (*n* = 15). In 9 domains, > 70% of stakeholders rated > 7, which the steering committee agreed (100% of votes, 15/15) was impractical to implement in clinical research. Instead, domains with > 80% of stakeholders rating > 7 were included as ‘essential’ components of the COS and those that had 70–80% of stakeholders rating > 7 were included as ‘recommended’ to measure. Measurement instruments were only assessed for the ‘essential’ domains.

Three domains were deemed ‘essential’ to include at 30 days (survival, physical function and infection) and five at 90 days (survival, physical function, activities of daily living, nutritional status and muscle/nerve function). A further four domains met criteria for ‘recommended’ to measure at 30 days (organ dysfunction, muscle/nerve function, nutritional status and wound healing) and three at 90 days (frailty, body composition and organ dysfunction). Table 1 summarises these data and more details are available in Additional file 1: Tables S8 and S9.

**Table 1:**
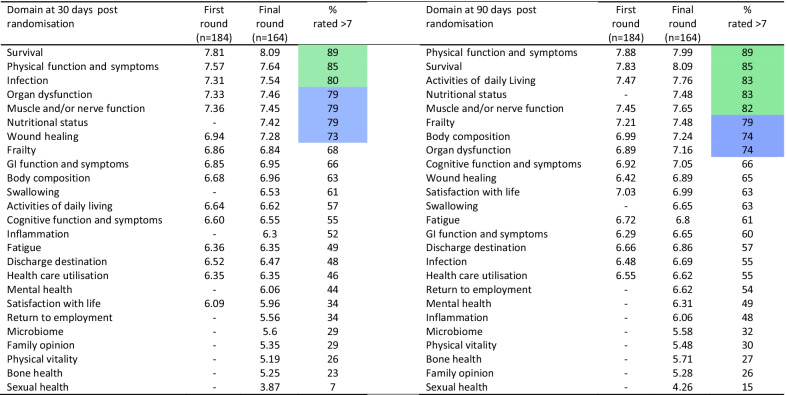
Domain performance across the Delphi rounds for 30 and 90 days post-randomisation

*Green* ‘essential’ components of the Core Outcome Set; *Blue* ‘recommended’ components

Data on the split of round 2 and the limited round 3 are available in the Additional file 1: Tables S8 and S9

### Measurement instruments at 30 days post-randomisation

The physical component score of the 36-item short form health survey (SF-36) was rated as critical to include (PCS; 75%, 80/107) [16]. Four measurement instruments scored between 60 and 70%: Administration of antibiotics (69%, 74/107), Sepsis 3.0 definition (67% 72/107), 30-s sit-to-stand (30STS; 65%, 70/107) and the 6-min walk test (6MWT; 62%, 66/107) [17–19]. Survival met criteria (93%, 42/45) as an ‘essential’ measure at the consensus meeting, with date of death relative to randomisation being agreed as a specific definition.

Concerns were raised during the consensus meeting in regard to mandating the PCS given the licence fee requirement, which would limit accessibility and therefore research in the field. Voting led to a consensus that it should be deemed ‘recommended’ as opposed to ‘essential’ (84%, 38/45). Regarding other instruments measuring the physical function domain, the 30STS met consensus criteria (92%, 46/50) for ‘recommendation’, but not the 6MWT (61%, 30/49). The suggested measurement instruments for the outcome domain of Infection did not meet criteria for ‘recommendation’ at 30 days post-randomisation: Administration of antibiotics (40%, 18/45) and Sepsis 3.0 definition (13%, 6/45). Measurement instrument data are summarised in Table 2 and Additional file 1: Table S12.

**Table 2:**
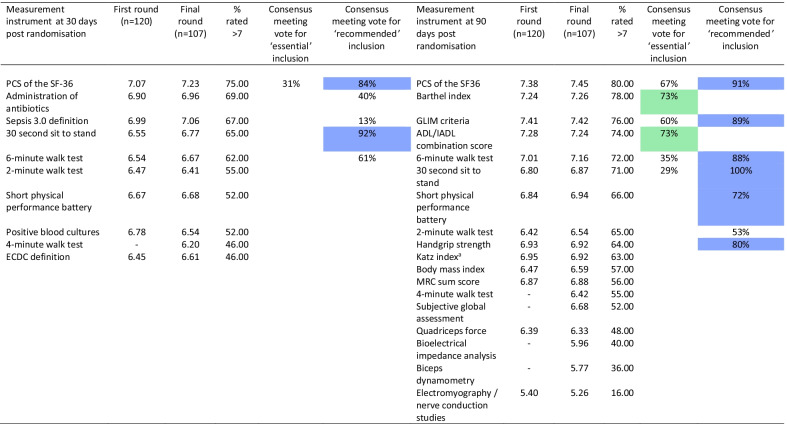
Measurement instrument performance across the Delphi rounds for 30 and 90 days post-randomisation and in the consensus meeting voting

*Green* ‘essential’ components of the Core Outcome Set; *Blue* ‘recommended’ components. *ADL* Activities of daily living; *ECDC* European centre for disease prevention and control; *GLIM* Global leadership initiative on malnutrition; *IADL* Instrumental activities of daily living; *MRC* Medical research council *PCS* Physical component score

More detail is available in the Additional file 1: Tables S12 and S14

^a^It was agreed at the consensus meeting that the Barthel or Katz indices could also be used as alternative to measure ADL

### Measurement instruments at 90 days post-randomisation

Prior to the consensus meeting, measurement instruments reaching ‘essential’ criteria were the PCS (80%, 87/107), Barthel Index (78%, 83/107), Global Leadership Initiative on Malnutrition (GLIM) criteria (76%, 81/107), Activities of daily living and Instrumental activities of daily living (ADL/IADL; 74%, 79/107), 6MWT (72%, 77/107) and the 30STS (71%, 76/107) [16, 18–22]. Instruments reaching 60–70% and therefore discussed at the consensus meeting were the Short Physical Performance Battery (SPPB; 66%, 71/107), the 2-min walk test (2MWT; 65%, 70/107), Handgrip strength (HGS; 64%, 68/107) and the Katz index (63%, 67/107) [23–26].

At the consensus meeting, date of death relative to randomisation achieved consensus for survival measurements (96%, 45/47). In the physical function domain, the PCS achieved consensus criteria for ‘recommendation’ (91%, 41/45) but not for ‘essential’ inclusion (67%, 33/49). The 30STS achieved 100% (40/40) consensus for ‘recommendation,’ having not met criteria for ‘essential’ inclusion (29%, 12/42). Similarly, the 6MWT achieved consensus for ‘recommendation’ (88%, 38/43) but not ‘essential’ inclusion (35%, 15/43). The SPPB achieved consensus for ‘recommendation’ (72%, 28/39), as did HGS (80%, 32/40), unlike the 2MWT (53%, 23/43).

Both ADL (73%, 24/33) and IADL (73%, 27/37) achieved consensus for ‘essential’ inclusion, and it was agreed that either the Barthel or Katz indices could be used as alternatives. Lastly the GLIM criteria achieved consensus for ‘recommendation’ (89%, 32/36) but not as ‘essential’ to include (60%, 21/35). Measurement instrument data are summarised in Table 2 and Additional file 1: Table S14.

The final COS is summarised in Table 3 and the process in Fig. 2.

**Table 3:** Summary table of the core outcome set for metabolic and nutritional outcomes interventions in critical illness

Essential	Measurement instruments	
Domain	30 days post-randomisation	90 days post-randomisation
Survival	Essential Day of death	Essential Day of death
Physical function	Essential - Recommended PCS SF-36 30STS	Essential - Recommended PCS SF-36 30STS 6MWT SPPB
Infection	Essential - Recommended -	
ADL/IADL		Essential Barthel/Katz or ADL/IADL Recommended -
Nutritional status		Essential - Recommended GLIM criteria
Muscle/Nerve function		Essential - Recommended HGS
	Recommended domains	
	Organ dysfunction	Organ dysfunction
	Muscle / nerve function	Frailty
	Nutritional status	Body composition
	Wound healing	

*30STS* 30 s sit-to-stand; *6MWT* 6-min walk test; *ADL* Activities of daily living; *GLIM* Global leadership on malnutrition; *HGS* Handgrip strength; *IADL* Instrumental activities of daily living; PCS *SF-36* Physical component score of the short form 36; *SPPB* Short physical performance battery

### Scoring by stakeholder group

*Domains* No difference was seen between groups for scoring of the domains at 30 days except for Nutritional status: clinical researchers vs. patients and caregivers (6.4 (2.1) vs. 7.3 (1.5); *p* = 0.037). At the 90 day time point, patients rated Activities of daily living and Body composition to be less important than healthcare professionals (7.3 (1.4) vs. 7.8 (1.1); *p* = 0.03) and (6.8 (1.3) vs. 7.6 (1.2); *p* = 0.001), respectively. Patients rated Organ dysfunction at 90 days to be of greater importance than both healthcare professionals (7.8 (1.2) vs.7.2 (1.7); *p* = 0.041) and clinical researchers (7.8 (1.2) vs. 6.6 (2.4); *p* = 0.013). The full breakdown of scores is shown in Additional file 1: Tables S10 and S11.

*Measurement instruments at 30 days post-randomisation* At 30 days, healthcare professionals rated the 6MWT (7.1 (1.6) vs. 6.1 (2.2); *p* = *0.04*) and antibiotic administration (7.2 (1.4) vs. 6.2 (1.9); *p* = 0.03) higher than clinical researchers. Healthcare professionals also rated the Sepsis 3.0 criteria lower than patients (7.2 (1.4) vs. 7.6 (1.2); *p* = 0.01). Researchers rated antibiotic administration (6.2 (2.9) vs. 7.6 (1.2); *p* = 0.002) and Sepsis 3.0 definition (6.6 (2.2) vs. 8.2 (1.2); *p* = 0.002) lower than patients and caregivers. Additional file 1: Table S13 details these differences.

*Measurement instruments at 90 days post-randomisation* At 90 days, healthcare professionals rated the 30STS (7.2 (1.2) vs. 6.3 (2.0); *p* = 0.05), 6MWT (7.5(1.2) vs. 6.5(2.0); *p* = 0.01) and GLIM criteria (7.7 (1.3) vs. 6.7 (2.1); *p* = 0.04) higher than clinical researchers. Healthcare professionals also rated HGS higher than patients (7.3 (1.4) vs. 6.4 (1.1); *p* = 0.006). Patients rated the SPPB higher than researchers (7.5 (1.0) vs. 6.3 (2.0); *p* = 0.009). Additional file 1: Table S15 details these differences.

## Discussion

We performed an international consensus process using a modified Delphi protocol, engaging with 213 patients, caregivers, healthcare professionals and clinical researchers from 24 countries. A consensus meeting with representatives from all stakeholder groups ensured the recommendations were as valid, feasible and accessible as possible, and that the psychometric properties of measurement tools had been robustly examined. Consensus was reached on domains and measurement instruments at two time points: 30 and 90 days from randomisation. Survival as determined by date of death from randomisation was the only common domain and measurement instrument that was determined ‘essential.’ Inter-stakeholder scoring variation was minimal, reflecting a strong consensus.

Given the breadth and scope of potential metabolic and nutritional interventions, it was perhaps unsurprising that no consensus could be reached on many of the proposed time points, nor that the threshold for inclusion had to be raised. Decisions were made therefore in the light of two guiding concepts. Firstly, that by using a fixed time point from randomisation, the statistical properties of the measurement tools may be easier to understand, enabling trial design, power calculations and data alignment of future trials to build an evidence base. It was also acknowledged that 30 days from randomisation may be at a similar time point to hospital discharge, and therefore, where in-hospital processes were relevant, hospital discharge may be an alternative time point albeit with different statistical properties. Secondly, there are an increasing number of COS being developed for critical illness, and all agreed that alignment with existing COS for longer-term outcomes would decrease duplication and increase external validity [12, 15].

### ‘Essential’ domains and measurement instruments

Physical function was an ‘essential’ domain at both time points, in keeping with the increasing focus on patient-centred outcomes. However, no measures were deemed ‘essential,’ reflecting three important points. Firstly, physical functional outcome research for metabolic and nutritional trials of critical illness remains an emerging field albeit of great interest to patients, researchers and funding bodies. There is a paucity of research in this field to inform confident decision-making in regard to mandating outcome assessment tools [7]. Secondly, post-hospital discharge follow-up research is difficult in critical illness survivors, and mandating measurement tools that require face-to-face interactions would be very challenging. Thirdly, the only tool meeting ‘essential’ criteria was the PCS of the SF-36, the psychometric properties being well established and appropriate across a range of comorbidities [15, 27]. The consensus meeting downgraded this to ‘recommended’ reflecting concerns about mandating a tool with a licence fee, limiting accessibility and therefore research in the field. An earlier RAND version of the SF-36 is, however, available without cost.

The 30STS met criteria and consensus for both time points. The STS is well defined and has been extensively used and its properties are examined across a wide spectrum of chronic diseases [28], with healthy age- and sex-matched data over normal ranges available [29]. This widespread use (including remotely [30]) and acceptability stems from the fundamental role that the ability to stand from sitting unaided has in ensuring independence of function and activities of daily living (e.g. getting out of bed, going to the toilet or getting up from a chair). Patients were especially taken with this measure, stating ‘*sit to stand is very straightforward, you simply count how many times you can fulfil the function in 30 s. Other than a dining room/kitchen chair no equipment is needed. It could be done in hospital or at home, over a video call. Any little improvements can mean a big deal in the early stages of recovery. Also you can’t fail, so even if you can only do it half a dozen times you still have something to record.*’ The 6MWT met consensus at 90 days, as concerns were raised regarding 30 days being too soon after the ICU episode. A shorter test, the 2MWT, did not achieve criteria for consensus at 30 days. A patient stated ‘*My concerns with the 6MWT is a patient's ability to do it. 30 days after ICU I don't think I could have done it, or it certainly would have been a struggle! This would have been demoralising, seeing it as failure, even if it wasn't. Any knock backs at this stage mean a lot more than usual, and are hard to rationalise.*’ In keeping with the focus on physical function, measures of ADL/IADL were deemed ‘essential’ at 90 days, though the heterogeneity of use and definition of IADL dependency was highlighted, again likely reflecting the paucity of data [31, 32]. Sixteen trials registered on ClinicalTrials.gov are planning to measure ADL/IADL, emphasising their current use. The Barthel or the Katz index remain acceptable alternatives until more data became available.

While the outcome of new infections was deemed important to measure, no outcome measure reached criteria for inclusion as ‘essential’ or ‘recommended.’ This was primarily as a result of the lack of certainty around the psychometric properties of the measurement instruments [33–35] and the routine empirical use of antibiotics [36]. Other COS for critically ill patients have included infection-related outcome measures, and inclusion of these might be more appropriate [12]. The GLIM scores met consensus for inclusion as a ‘recommended’ measurement. It was noted that the GLIM criteria were designed to be as broad as possible and have been utilised post-hospital discharge in observational studies, though subjectivity in scoring may be a clinimetric limitation [37].

### Other ‘recommended’ domains

Organ dysfunction met criteria for ‘recommendation’ to measure at both time points. ‘Recommended’ domains were not taken forward into the Delphi consensus for measurement instruments, given the number of domains deemed ‘essential’ to measure. Organ dysfunction, like frailty (‘recommended’ at 90 days) has not been a well-used outcome measure in nutritional and metabolic trials, with a few notable exceptions, and more data are required both on potential measurement instruments and their psychometric properties [38]. Body composition, muscle/nerve function and nutritional status have traditionally been used either as primary or secondary outcome measures in metabolic and nutritional trials outside critical illness. The shift of these away from ‘essential’ to ‘recommended’ outcome domains for the critically ill population implies a greater weight being given to functional, patient-centred outcomes.

### Strengths and limitations

This COS has several strengths, notably the high level of engagement internationally from clinicians and academics (24 countries), and the patients and caregivers taking part in the Delphi process represented 6 countries, helping support external validity. The composition of the panel and the suggestions regarding outcomes are open to bias as are all expert consensus processes, especially from clinical researchers with potential conflict of interests. This may have resulted in certain outcomes not being included in the consensus process, although participants were able to suggest additional domains and instruments, and consensus was reached on multiple domains and instruments with modest variability between stakeholder groups. Following stage 1 of the Delphi, we divided the domains reaching consensus criteria into ‘recommended’ and ‘essential’ groups. This was due to the large number of domains with high scores compromising the utility and feasibility of the final COS. As this was a change to the original methodology there is a risk of bias. This was minimised by using voting which had been agreed a priori. A well-known limitation in Delphi methodology is attrition of participants between rounds; however, in the Delphi the response rates were universally > 80% when compared to the previous round, which is considered satisfactory [39]. A major limitation remains the evidence base for longitudinal outcome measures in the critical illness survivor cohort, an issue that has been raised in the development of other COS [15]. Unique to this COS is the heterogeneity of interventions that are likely to be assessed, widening the field of outcomes. No biological endpoints met criteria for inclusion, though the microbiome and inflammation were put forward in the initial and second rounds. This reflects again the increasing prominence of patient-centred outcomes, and the current lack of clear relationship these biological markers has with said outcomes. These recommendations and the standardisation of time points will contribute infrastructure to the development of this evidence base, to inform a future update of this COS.

### Conclusions

Metabolic and nutritional interventional research in critically ill patients has increasingly focused on physical functional outcomes. We recommend the CONCISE COS derived in this study, an internationally agreed minimum set of outcomes, for use at 30 and 90 days post-randomisation in all clinical research focusing on nutritional and metabolic interventions.

## Supplementary Information

**Additional file 1**. Supplementary methods, results, figures and tables.

## Data Availability

The datasets used and/or analysed during the current study are available from the corresponding author on reasonable request.
