# Supplementary material for: Core outcome measures for clinical effectiveness trials of nutritional and metabolic interventions in critical illness: an international modified Delphi consensus study evaluation (CONCISE)
Source: Crit Care. 2022 Aug 6;26:240. doi: 10.1186/s13054-022-04113-x (PMC9357332; doi:10.1186/s13054-022-04113-x)
Supplement: Supplementary file 1 — Additional file 1. Supplementary methods, results, figures and tables. [file 13054_2022_4113_MOESM1_ESM.docx]

**Core Outcome Measures for Clinical Effectiveness Trials of Nutritional and Metabolic Interventions in Critical Illness: An International Modified Delphi Consensus Study Evaluation (CONCISE) - SUPPLEMENTARY INFORMATION**

Davies TW^1,2^, van Gassel RJJ^3,4^, van de Poll M^3,4^, Gunst, J^5^, Casaer MP^5^, Christopher KB^6^, Preiser JC^7^, Hill A^8^, Gundogan K^9^, Reintam-Blaser A^10,11^, Rousseau A.-F.^12^, Hodgson C^13,14^, Needham DM^15,16^, Castro M^17^, Schaller S^18,19^, McClelland T^1,2^, Pilkington JJ^20^, Sevin CM^21^, Wischmeyer PE^22^, Lee ZY^23^, Govil D^24^, Li A^25,26^, Chapple L^27^, Denehy L^28,29^, Montejo-González JC^30^, Taylor B^31^, Bear DE^32^, Pearse R^1,2^, McNelly A^1^, Prowle J^1,2^, Puthucheary ZA^1,2^

^1^William Harvey Research Institute, Barts and The London School of Medicine & Dentistry, Queen Mary University of London

^2^Adult Critical Care Unit, Royal London Hospital, London

^3^ Department of Intensive Care Medicine, School of Nutrition and Translational Research in Metabolism (NUTRIM), Maastricht University Medical Centre+, Maastricht, The Netherlands

^4^Department of Surgery, School of Nutrition and Translational Research in Metabolism (NUTRIM), Maastricht University Medical Centre+, Maastricht, The Netherlands

^5^Clinical Department and Laboratory of Intensive Care Medicine, Department of Cellular and Molecular Medicine, KU Leuven, Herestraat 49, 3000, Leuven, Belgium

^6^Division of Renal Medicine, Channing Division of Network Medicine, Brigham and Women's Hospital, USA

^7^Medical Direction, Erasme University Hospital, Universite Libre de Bruxelles, Brussels, Belgium

^8^Departments of Intensive Care and Anesthesiology, University Hospital RWTH Aachen University, D-52074 Aachen, Germany.

^9^Division of Intensive Care Medicine, Department of Internal Medicine, Erciyes University School of Medicine, Kayseri, Turkey

^10^Department of Anaesthesiology and Intensive Care, University of Tartu,Tartu, Estonia

^11^Department of Intensive Care Medicine, Lucerne Cantonal Hospital, Lucerne, Switzerland

^12^Department of Intensive Care, University Hospital of Liège, Liege, Belgium

^13^Australian and New Zealand Intensive Care Research Centre, School of Public Health and Preventive Medicine, Monash University, 3/553 St Kilda Rd, Melbourne, VIC, 3004, Australia

^14^Department of Intensive Care and Hyperbaric Medicine, The Alfred, Melbourne, VIC, Australia

^15^Outcomes After Critical Illness and Surgery (OACIS) Research Group, Johns Hopkins University, Baltimore, MD, USA

^16^Pulmonary and Critical Care Medicine, Department of Medicine, and Department of Physical Medicine and Rehabilitation Johns Hopkins University School of Medicine, Baltimore, MD, USA

^17^Clinical Nutrition, Hospital Israelita Albert Einstein, Sao Paulo, Brazil

^18^Charité - Universitätsmedizin Berlin, Corporate member of Freie Universität Berlin, Humboldt-Universität zu Berlin, Berlin Institute of Health, Department of Anesthesiology and Operative Intensive Care Medicine (CVK, CCM), Berlin, Germany

^19^Technical University of Munich, School of Medicine, Klinikum rechts der Isar, Department of Anesthesiology and Intensive Care, Munich, Germany

^20^Centre for Bioscience, Manchester Metropolitan University, John Dalton Building, Chester Street, Manchester, UK

^21^Department of Medicine, Division of Allergy, Pulmonary, and Critical Care Medicine, Vanderbilt University Medical Center, Nashville, TN

^22^Department of Anesthesiology, Duke University School of Medicine, DUMC, Box 3094 Mail # 41, 2301 Erwin Road, 5692 HAFS, Durham, NC, NC 27710, USA

^23^Department of Anesthesiology, University of Malaya, Kuala Lumpur, Malaysia

^24^Institute of Critical Care and Anesthesia, Medanta: The Medicty, Gurugram, Haryana, India

^25^Division of Respiratory and Critical Care Medicine, Department of Medicine, National University Hospital, National University Health System, Singapore

^26^Department of Intensive Care Medicine, Woodlands Health, Singapore

^27^Adelaide Medical School, Faculty of Health and Medical Sciences, The University of Adelaide, Adelaide, South Australia, Australia

^28^The University of Melbourne, School of Health Sciences, Melbourne, Australia

^29^Department of Allied Health, Peter McCallum Cancer Centre, Melbourne, Australia

^30^Department of Intensive Care Medicine, Hospital Universitario 12 de Octubre, Madrid, Spain

^31^Department of Research for Patient Care Services, Barnes-Jewish Hospital, St. Louis, Missouri, USA

^32^Guy´s and St Thomas’ NHS Foundation Trust, Department of Critical Care and Department of Nutrition and Dietetics, London, United Kingdom

***Methods***

***Systematic Review***

*Title*

Nutritional and metabolic treatment outcomes in critical care: an updated systematic review of randomised controlled trials

*Protocol*

This review is an update of a previously published review [1]. It is a systematic review to identify the outcomes measures used in recently published randomised controlled trials (RCTs) of nutritional interventions in the critically ill population. The Preferred Items for Systematic Reviews and Meta-Analyses (PRISMA) reporting guidelines were followed (Supplementary Information (SI) Table S1), and it was prospectively registered on PROSPERO (CRD42021242457).

Relevant RCTs published between September 2018 and March 2021 were identified by electronically searching MEDLINE (31^st^ August 2018 – 21^st^ March 2021) and the Cochrane Library) (2018-2021). The following MeSH and free text terms were used “nutrition”, “feeding”, “alimentation” “protein-calorie”, “nutritional support”, “critical care”, “critically ill”, “critical illness”, “intensive care”, “ICU”, “adult”, “randomized controlled trial”, “controlled clinical trial”, “randomized”, and “trial”. The search was limited to articles in English. The bibliographies of included studies were scanned, and experts consulted to identify studies that were missed by the search. Unpublished relevant RCTs were identified by searching the ClinicalTrials.gov registry using the following criteria: adult or older adult; start date on or after 31 August 2018; interventional studies (clinical trials); and the key words nutrition, feeding, protein-calorie, nutritional support, critical care, critically ill, intensive care, ICU, critical illness, and randomized.

We extracted records to Mendeley (London, UK) to sort and remove duplicates. Two investigators (TWD and RVG) independently reviewed each record by title and abstract. Papers identified as potentially relevant were reviewed in full. Papers were selected for inclusion if they were an RCT, including critically ill adults (age > 18 years), evaluating a nutritional intervention, and published between August 31st 2018 and March 21^st^ 2021. We did not include neonatal or paediatric intensive care studies, studies focusing on the pharmacological properties of enteral nutrition (EN) or parenteral nutrition (PN) without supplying details on nutritional support, abstracts, case reports, review articles and ancillary studies (sub analysis or separate from main RCT). Differences in opinion were resolved through discussion and referred to a third investigator (ZP).

One author (TWD) developed the first draft of the data extraction form to gather information on the items of interest. This was reviewed by another author (JJP) and revised after discussion. Data were extracted by two of us (TWD and JJP) who worked independently of each other. The following data were recorded: article characteristics (title, lead author, year of publication); study characteristics (population, study design, blinding method, source of funding, number of centres, and geographic region, number of patients randomised, duration of intervention, length of time to primary outcome measurement in days, outcome lag time); main study topic, recorded as clinical nutrition strategy, composition of nutritional support, or nutritional supplementation; and primary and secondary outcomes. Outcomes were classified using predefined categories including mortality (intensive care unit (ICU) mortality, in-hospital mortality, day 28 mortality, day 29-89 mortality, day 90 mortality, day>90 mortality), length of stay (in the ICU and in hospital), duration of organ dysfunction (time on mechanical ventilation, vasopressor/inotrope infusion, renal replacement therapy; organ failure; and antibiotic therapy), complications (infections, metabolic complications, feeding intolerance), functional outcomes during the study period (muscle strength, walking distance tests, quality of life, physical function), and other (metabolic concentration, feeding measures, tube placement (time needed to place or success rate), contamination, muscle mass, discharge location, nitrogen balance). All data were extracted and populated into a Google Sheets database (Google, CA, USA).

*PRISMA Checklist*

**Supplementary Information Table S1 PRISMA Checklist**

| **Section and Topic** | **Item #** | **Checklist item** | **Location where item is reported** |
| --- | --- | --- | --- |
| **TITLE** | | |  |
| Title | 1 | Identify the report as a systematic review. | SI |
| **ABSTRACT** | | |  |
| Abstract | 2 | See the PRISMA 2020 for Abstracts checklist. | N/A |
| **INTRODUCTION** | | |  |
| Rationale | 3 | Describe the rationale for the review in the context of existing knowledge. | PROSPERO |
| Objectives | 4 | Provide an explicit statement of the objective(s) or question(s) the review addresses. | PROSPERO |
| **METHODS** | | |  |
| Eligibility criteria | 5 | Specify the inclusion and exclusion criteria for the review and how studies were grouped for the syntheses. | Protocol in SI |
| Information sources | 6 | Specify all databases, registers, websites, organisations, reference lists and other sources searched or consulted to identify studies. Specify the date when each source was last searched or consulted. | Protocol in SI |
| Search strategy | 7 | Present the full search strategies for all databases, registers and websites, including any filters and limits used. | Protocol in SI |
| Selection process | 8 | Specify the methods used to decide whether a study met the inclusion criteria of the review, including how many reviewers screened each record and each report retrieved, whether they worked independently, and if applicable, details of automation tools used in the process. | Protocol in SI |
| Data collection process | 9 | Specify the methods used to collect data from reports, including how many reviewers collected data from each report, whether they worked independently, any processes for obtaining or confirming data from study investigators, and if applicable, details of automation tools used in the process. | Protocol in SI |
| Data items | 10a | List and define all outcomes for which data were sought. Specify whether all results that were compatible with each outcome domain in each study were sought (e.g. for all measures, time points, analyses), and if not, the methods used to decide which results to collect. | Protocol in SI |
|  | 10b | List and define all other variables for which data were sought (e.g. participant and intervention characteristics, funding sources). Describe any assumptions made about any missing or unclear information. | Protocol in SI |
| Study risk of bias assessment | 11 | Specify the methods used to assess risk of bias in the included studies, including details of the tool(s) used, how many reviewers assessed each study and whether they worked independently, and if applicable, details of automation tools used in the process. | SI |
| Effect measures | 12 | Specify for each outcome the effect measure(s) (e.g. risk ratio, mean difference) used in the synthesis or presentation of results. | N/A |
| Synthesis methods | 13a | Describe the processes used to decide which studies were eligible for each synthesis (e.g. tabulating the study intervention characteristics and comparing against the planned groups for each synthesis (item #5)). | SI |
|  | 13b | Describe any methods required to prepare the data for presentation or synthesis, such as handling of missing summary statistics, or data conversions. | N/A |
|  | 13c | Describe any methods used to tabulate or visually display results of individual studies and syntheses. | SI |
|  | 13d | Describe any methods used to synthesize results and provide a rationale for the choice(s). If meta-analysis was performed, describe the model(s), method(s) to identify the presence and extent of statistical heterogeneity, and software package(s) used. | N/A |
|  | 13e | Describe any methods used to explore possible causes of heterogeneity among study results (e.g. subgroup analysis, meta-regression). | N/A |
|  | 13f | Describe any sensitivity analyses conducted to assess robustness of the synthesized results. | N/A |
| Reporting bias assessment | 14 | Describe any methods used to assess risk of bias due to missing results in a synthesis (arising from reporting biases). | N/A |
| Certainty assessment | 15 | Describe any methods used to assess certainty (or confidence) in the body of evidence for an outcome. | N/A |
| **RESULTS** | | |  |
| Study selection | 16a | Describe the results of the search and selection process, from the number of records identified in the search to the number of studies included in the review, ideally using a flow diagram. | CONSORT Diagram in SI |
|  | 16b | Cite studies that might appear to meet the inclusion criteria, but which were excluded, and explain why they were excluded. | N/A |
| Study characteristics | 17 | Cite each included study and present its characteristics. | SI Table S1 in SI |
| Risk of bias in studies | 18 | Present assessments of risk of bias for each included study. | SI Figure S3 in SI |
| Results of individual studies | 19 | For all outcomes, present, for each study: (a) summary statistics for each group (where appropriate) and (b) an effect estimate and its precision (e.g. confidence/credible interval), ideally using structured tables or plots. | SI |
| Results of syntheses | 20a | For each synthesis, briefly summarise the characteristics and risk of bias among contributing studies. | SI |
|  | 20b | Present results of all statistical syntheses conducted. If meta-analysis was done, present for each the summary estimate and its precision (e.g. confidence/credible interval) and measures of statistical heterogeneity. If comparing groups, describe the direction of the effect. | SI |
|  | 20c | Present results of all investigations of possible causes of heterogeneity among study results. | N/A |
|  | 20d | Present results of all sensitivity analyses conducted to assess the robustness of the synthesized results. | N/A |
| Reporting biases | 21 | Present assessments of risk of bias due to missing results (arising from reporting biases) for each synthesis assessed. | N/A |
| Certainty of evidence | 22 | Present assessments of certainty (or confidence) in the body of evidence for each outcome assessed. | N/A |
| **DISCUSSION** | | |  |
| Discussion | 23a | Provide a general interpretation of the results in the context of other evidence. | Main manuscript |
|  | 23b | Discuss any limitations of the evidence included in the review. | Limitations in SI |
|  | 23c | Discuss any limitations of the review processes used. | Limitations in SI |
|  | 23d | Discuss implications of the results for practice, policy, and future research. | Main manuscript |
| **OTHER INFORMATION** | | |  |
| Registration and protocol | 24a | Provide registration information for the review, including register name and registration number, or state that the review was not registered. | Protocol in SI |
|  | 24b | Indicate where the review protocol can be accessed, or state that a protocol was not prepared. | SI |
|  | 24c | Describe and explain any amendments to information provided at registration or in the protocol. | N/A |
| Support | 25 | Describe sources of financial or non-financial support for the review, and the role of the funders or sponsors in the review. | Main manuscript |
| Competing interests | 26 | Declare any competing interests of review authors. | Main manuscript |
| Availability of data, code and other materials | 27 | Report which of the following are publicly available and where they can be found: template data collection forms; data extracted from included studies; data used for all analyses; analytic code; any other materials used in the review. | N/A |

SI = supplementary information; N/A = not applicable

*CONSORT Diagram*

**Supplementary Information Fig S1 Record extraction flowchart for systematic review**


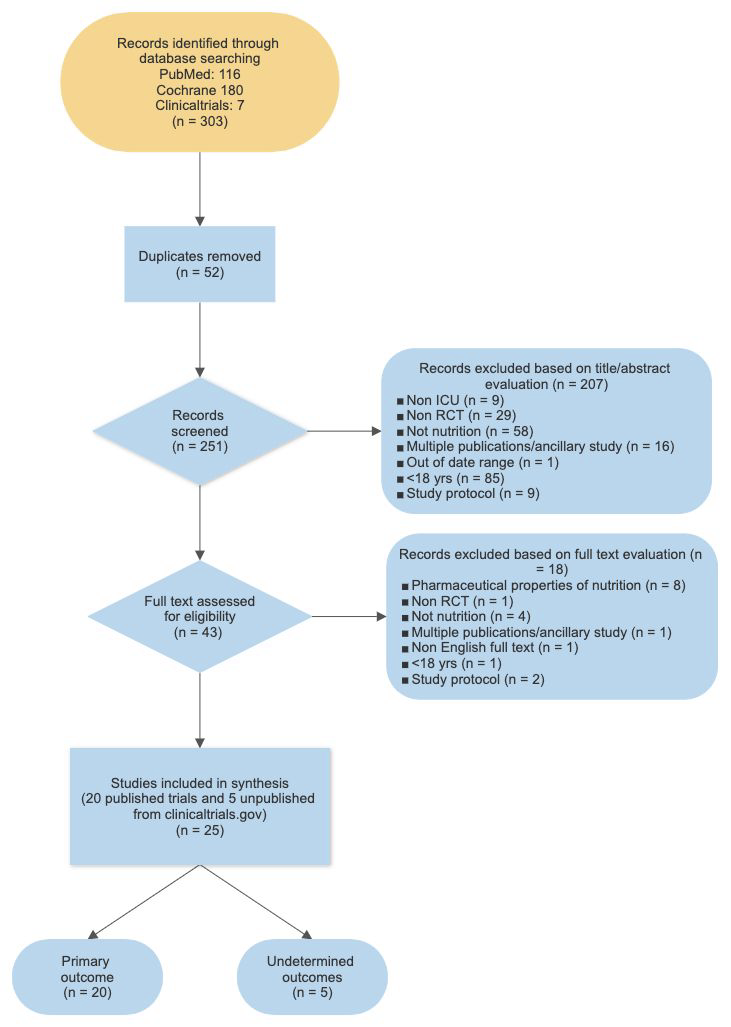


ICU = intensive care unit; RCT = randomised controlled trial

***Steering Committee Members***

**Supplementary Information Table S2 Steering Committee Members**

| **Steering Committee Representative** | **Country** | **Job Role** |
| --- | --- | --- |
| Aileen Hill | Germany | Clinician Scientist, Intensivist and Nutritionist |
| Andrew Li | Singapore | Associate Consultant in Critical Care |
| Angela McNelly | United Kingdom | Post Doctoral Fellow |
| Anne-Françoise Rousseau | Belgium | Intensivist and Assistant Lecturer |
| Annika Reintam Blaser | Switzerland | Senior Critical Care Consultant and Associate Professor |
| Beth Taylor | USA | Dietitian and Research Scientist |
| Carla Sevin | USA | Intensivist and Assistant Professor |
| Carol Hodgson | Australia | Professor of ICU Research and Specialist Intensive Care Physio |
| Dale Needham | USA | Professor of Pulmonary & Critical Medicine, and Physical Medicine and Rehabilitation |
| Danielle Bear | United Kingdom | Dietitian |
| Deepak Govil | India | Director of Critical Care |
| Jan Gunst | Belgium | Associate Professor Intensive Care |
| Jean Charles Preiser | Belgium | Medical Director of Research |
| Juan Carlos Montejo-González | Spain | Intensive Care Doctor and Head of Unit |
| Kenneth Christopher | USA | Assistant Professor |
| Kursat Gundogan | Turkey | Professor of Intensive Care |
| Lee-anne Chapple | Australia | Clinical Dietitian and Post Doctoral Fellow |
| Linda Denehy | Australia | Professor of Physiotherapy |
| Marcel van de Poll | Netherlands | Assistant Professor and Intensivist |
| Melina Castro | Brazil | Intensive Care Doctor and Researcher |
| Michael Casaer | Belgium | Professor of Intensive care |
| Paul Wischmeyer | USA | Professor of Anaesthesiology and Surgery |
| Rob van Gassel | Netherlands | Clinician and PhD Candidate |
| Stefan Schaller | Germany | Professor of Intensive Care and Deputy Clinical Director |
| Thomas Davies | United Kingdom | Clinician and Academic Clinical Fellow |
| Zheng Yii Lee | Malaysia | Dietitian and Post Doctoral Fellow |
| Zudin Puthucheary | United Kingdom | Intensive Care Consultant and Senior Lecturer |

***Systematic Review - Outcomes measured and extracted for Delphi***

Supplementary Information Table S3 Breakdown of outcome measures for randomised controlled trials

| Outcome | Primary^a^  n=20/25 | Secondary^b^  n=20/25 | Undetermined^a^  n=5/25 |
| --- | --- | --- | --- |
| Mortality, n(%)  ICU  Hospital  Day 28  Day 29-89  Day 90  Day 90+ | 3 (15)  0  1  1  0  2  0 | 14 (70)  6  8  4  4  1  2 | 4 (80)  2  1  1  1  0  0 |
| Length of stay n(%)  ICU  Hospital | 1(5)  1  1 | 14 (70)  12  13 | 4 (80%)  4  2 |
| Organ Dysfunction, n(%)  Mechanical Ventilation  Vasopressor/inotrope infusion  Renal Replacement Therapy  Organ failure  Antibiotic therapy | 1(5)  1  0  0  0  0 | 15(75)  14  2  5  4  2 | 3 (60)  3  0  0  3  0 |
| Complications, n(%)  Infections  Metabolic  Feeding intolerance  Skin alterations | 3 (15)  2  0  1  0 | 15 (75)  5  3  13  1 | 3 (60)  1  1  3  0 |
| Functional Outcomes, n (%)  Quality of Life  Physical function  Muscle strength  Walking distance  Activities of daily living | 3 (15)  1  1  0  1  0 | 8 (40)  6  6  6  2  0 | 2 (10)  0  0  1  0  0 |
| Others (n%)  Metabolic concentration^b^  Feeding measures^c^  Muscle mass  Tube placement  Equipment contamination  Nitrogen balance  Discharge location | 9 (45)  0  5  4  0  0  1  0 | 18 (90)  10  10  6  0  0  0  3 | 5 (100)  5  4  0  0  0  1  0 |

n(%) refers to number and percentage of trials. ^a^Most studies had more than one secondary and/or undetermined outcomes. ^b^Metabolic concentration was defined as any outcome relevant to the blood level of a substance. ^c^Feeding measures were all outcomes directly related to the nutritional intervention e.g. calories or protein delivered. ICU = intensive care unit

***Delphi panel participants***

Inclusion criteria: Clinical researchers and healthcare professionals with experience of working and research within the critical care area: any gender; 18-99 years. Patients and caregivers who have experienced an ICU stay, either themselves or amongst family members or friends: any gender; 18-99 years, and access to the internet.

**Supplementary Information Table S4 Delphi panel participants**

| **Continent, Country, Stakeholder Group** | **Representative** | **Clinical Role** | **Academic Role** |
| --- | --- | --- | --- |
| **Africa** |  |  |  |
| **Egypt** |  |  |  |
| *Healthcare Professionals* |  |  |  |
|  | Mohamed Elbahnasawy |  |  |
| **Morocco** |  |  |  |
| *Clinical Researchers* |  |  |  |
|  | Ghannam Abdelilah | Intensivist | Professor |
| **Asia** |  |  |  |
| **Malaysia** |  |  |  |
| *Clinical Researchers* |  |  |  |
|  | Mazuin Kamarul Zaman | Dietitian | Lecturer |
|  | Sivanesan Subramanian |  |  |
|  | Lee Zheng Yii | Dietitian | Researcher |
| *Healthcare Professionals* |  |  |  |
|  | Cindy Sing Ling Yap | Dietitian |  |
|  | Wong Wei Jin | Surgeon |  |
|  | Mohd Basri Mat Nor | Senior Consultant Intensivist | Professor |
|  | Pui Hing Foong | Dietitian |  |
|  | Jasreena Gill | Pharmacist |  |
| *Patients or Caregivers* |  |  |  |
|  | Au Yong Heng Quen |  |  |
|  | David K T Chua |  |  |
|  | Izham Bin Abdul Rahim |  |  |
| **Philippines** |  |  |  |
| *Healthcare Professionals* |  |  |  |
|  | Grace Paguia | Physician |  |
| **Singapore** |  |  |  |
| *Clinical Researchers* |  |  |  |
|  | Will Loh | Intensive Care Consultant | Research Director |
| *Healthcare Professionals* |  |  |  |
|  | Kollengode Ramanathan | Intensive Care Specialist |  |
|  | Charles Chin Han Lew | Dietitian |  |
|  | Alvin Wong | Clinical Dietitian |  |
|  | Cherie Tong | Dietitian |  |
|  | Andrew Li | Physician |  |
| **Turkey** |  |  |  |
| *Clinical Researchers* |  |  |  |
|  | Kursat Gundogan | Intensive Care | Professor |
|  | Hilal Sipahioglu | Doctor | Researcher |
|  | Meltem Şimşek | Medical Doctor | Researcher |
|  | Fatma Yildirim | Researcher | Associate Professor |
|  | Avşar Zerman | Doctor | Assistant Professor |
|  | Emre Aydin |  |  |
| *Healthcare Professionals* |  |  |  |
|  | Gulbin Aygencel | Intensivist |  |
|  | Türkay Akbaş | Intensivist |  |
|  | Neriman Defne Altintas | Head of Division of Medical Intensive Care |  |
|  | Murat Sungur | Doctor |  |
|  | Kubilay Demirağ | Intensivist |  |
|  | Kemal Tolga Saracoglu | Head of Department of Anesthesiology |  |
|  | Şahin Temel | Critical Care |  |
|  | Pervin Hancı | Intensivist |  |
|  | Burcu | Clinical Pharmacist |  |
|  | Nazlihan Boyaci Dundar | Intensive Care Specialist |  |
| **Australia** |  |  |  |
| **Australia** |  |  |  |
| *Clinical Researchers* |  |  |  |
|  | Emma Ridley | Senior Dietitian | Senior Research Fellow |
|  | Anne Leditschke | Intensivist | Associate Professor |
|  | Andrea Marshall | Registered Nurse | Professor |
|  | Lewis Campbell | Intensivist | Associate Professor |
|  | Marianne Chapman | Intensivist | Professor |
|  | Adam M Deane | Intensivist | Associate Professor |
| *Healthcare Professionals* |  |  |  |
|  | Brydie Cleeve | Dietitian |  |
|  | Adrian Regli | Consultant |  |
|  | Caroline Guille | Manager Of Dietetics |  |
|  | Jacinta Winderlich | Paediatric Dietitian |  |
|  | Clare Ferguson | Clinical Dietitian |  |
|  | Greta Hollis | Intensive Care Dietitian |  |
| **New Zealand** |  |  |  |
| *Clinical Researchers* |  |  |  |
|  | Varsha Asrani | Advanced Clinician: Intensive Care Dietitian | PhD Research Fellow |
|  | John Windsor | Surgeon | Professor |
| *Healthcare Professionals* |  |  |  |
|  | Rebecca Baskett | Intensive Care Dietitian |  |
| **Europe** |  |  |  |
| **Austria** |  |  |  |
| *Clinical Researchers* |  |  |  |
|  | Michael Hiesmayr | Senior Researcher | Professor |
| **Belgium** |  |  |  |
| *Clinical Researchers* |  |  |  |
|  | Jean Charles Preiser | Medical Director of Research | Professor |
|  | Jan Gunst | Consultant Intensivist | Associate Professor |
|  | Anne-Françoise Rousseau | Intensivist | Assistant Lecturer |
|  | Elisabeth De Waele | Intensivist | Assistant Professor |
|  | Rik Gosselink | Physiotherapist | Professor Rehabilitation Sciences |
|  | Michael Casaer | Intensive Care Faculty & Researcher | Professor |
| *Healthcare Professionals* |  |  |  |
|  | Anne-Marie Verbrugge | Dietician |  |
|  | Karolien Dams | Senior Staff Member Intensive Care |  |
|  | Didier Ledoux | Intensive Care Physician |  |
|  | Vincent Fraipont | Intensivist-Internist |  |
|  | Xavier Wittebole | Critical Care Physician |  |
| *Patients or Caregivers* |  |  |  |
|  | Jocelyne Puts |  |  |
|  | Philippe Vilenne |  |  |
|  | Brigitte Requiere |  |  |
| **Estonia** |  |  |  |
| *Clinical Researchers* |  |  |  |
|  | Alastair Forbes | Gastro/Nutrition | Professor |
| *Healthcare Professionals* |  |  |  |
|  | Kadri Tamme | Intensive Care Physician |  |
| **France** |  |  |  |
| *Clinical Researchers* |  |  |  |
|  | Pierre Garcon | Intensive Care Doctor | Doctor |
| *Healthcare Professionals* |  |  |  |
|  | Ronan Thibault | Doctor |  |
| **Germany** |  |  |  |
| *Clinical Researchers* |  |  |  |
|  | Gunnar Elke | Consultant Anaesthesia/Critical Care | Associate Professor |
|  | Steffen Weber-Carstens | Leading Consultant Intensive Care | Professor |
|  | Andreas Edel | Consultant |  |
|  | Stefan Schaller | Deputy Clinical Director | Professor |
|  | Arved Weimann | Surgeon | Professor |
|  | Christian Stoppe | Consultant Critical Care Medicine And Trialist | Clinician Scientist |
| *Healthcare Professionals* |  |  |  |
|  | Sebastian Fritsch | Physician |  |
|  | Geraldine De Heer | Senior Consultant |  |
|  | Dorothea Puchstein |  |  |
|  | Ak Krueger | Anesthesiologist |  |
|  | Wolfgang Hartl | Director Sicu |  |
|  | Aileen Hill | Resident Intensive Care |  |
| **Italy** |  |  |  |
| *Clinical Researchers* |  |  |  |
|  | Nicola Latronico | Director/Doctor | Professor |
| **Netherlands** |  |  |  |
| *Clinical Researchers* |  |  |  |
|  | Rob van Gassel | Clinician | PhD Candidate |
| *Healthcare Professionals* |  |  |  |
|  | Ben van der Hoven | Intensivist |  |
|  | Albertus Beishuizen | Intensivist |  |
|  | Eva Grimbergen | Dietitian |  |
|  | Marcel van de Poll | Intensivist |  |
|  | Karen Ottens-Oussoren | Dietitian |  |
|  | Arthur van Zanten | Intensivist |  |
| *Patients or Caregivers* |  |  |  |
|  | J. Valentijn |  |  |
|  | Marjolein Siebel |  |  |
|  | Tammo Oegema |  |  |
| **Russia** |  |  |  |
| *Healthcare Professionals* |  |  |  |
|  | Mikhail Kirov | Professor |  |
| *Patients or Caregivers* |  |  |  |
|  | Vadim A. Mazurok |  |  |
| **Serbia** |  |  |  |
| *Healthcare Professionals* |  |  |  |
|  | Jovana Stanisavljevic | Specialist In Anesthesiology and Intensive Care |  |
| **Spain** |  |  |  |
| *Clinical Researchers* |  |  |  |
|  | Sergio Ruiz-Santana | Intenisve Care Chairman | Full Professor of Medicine |
| *Healthcare Professionals* |  |  |  |
|  | Luisa Bordejé Laguna | Doctor |  |
|  | Julai Alvarez Hernandez | Endocrinology and Nutrition Specialist |  |
|  | José Luis Flordelís Lasierra | Intensive Care Medicine Physician |  |
|  | Maria Gero Escapa | Critical Care Medical Specialist |  |
|  | Abelardo Garcia-De-Lorenzo | Head Of Intensive Care Medicine Service |  |
|  | Clara Vaquerizo | Medical Doctor (Intensivist) |  |
|  | Juan C Montejo | Doctor and Head of Unit |  |
| **Switzerland** |  |  |  |
| *Clinical Researchers* |  |  |  |
|  | Annika Reintam Blaser | Senior Consultant | Associate Professor |
| *Healthcare Professionals* |  |  |  |
|  | Boutaina Zemrani | Paediatrician and Specialist in Nutrition |  |
| *Patients or Caregivers* |  |  |  |
|  | Joppe Jansen |  |  |
| **United Kingdom** |  |  |  |
| *Clinical Researchers* |  |  |  |
|  | Zudin Puthucheary | Intensive Care Consultant | Senior Lecturer |
| *Healthcare Professionals* |  |  |  |
|  | Louise Albrich | Advanced Dietitian (Critical Care) |  |
|  | Lauren Fixter | Intensive Care Dietitian |  |
|  | Ella Terblanche | Dietitian |  |
|  | Nitin Arora | Consultant Intensivist |  |
|  | Tamara | Dietitian |  |
|  | Georgia Hardy | Dietitian |  |
|  | Rebecca Kirby | Dietitian |  |
|  | Rebecca Youngman | Dietitian |  |
|  | Robert Cronin | Dietitian |  |
|  | Nicky Wyer | Dietitian |  |
|  | Danielle Bear | Dietitian |  |
|  | Alice Extance | Dietitian |  |
|  | James Jackson | Dietitian |  |
|  | Tom Hollis | Dietitian |  |
|  | Bethan Jenkins | Dietitian |  |
|  | Robert Wise | Intensive Care Consultant |  |
|  | Jessica Zekavica | Dietitian |  |
|  | Katy Stuart | Critical Care Dietitian |  |
|  | Quynh Thi Nhu Truong | Critical Care Dietitian |  |
|  | Saira Khan | Dietitian |  |
|  | Felicity Gove | Dietitian |  |
|  | Emma Gaskin | Dietitian |  |
|  | Luke Cunningham | Dietitian |  |
|  | Jaci Chapman | Critical Care Dietitian |  |
| *Patients or Caregivers* |  |  |  |
|  | Shaji Joseph |  |  |
|  | Donna Reid |  |  |
|  | Mark Hudson |  |  |
|  | Lynn Simpson |  |  |
|  | Mr Ian J Wilson |  |  |
|  | Sally Andrews |  |  |
|  | Dr Smarajit Roy |  |  |
|  | John Wilson |  |  |
|  | Francesco Palma |  |  |
|  | Sarifa Patel |  |  |
|  | Rebecca Langley |  |  |
|  | Patrick Behan |  |  |
|  | Casey Coats |  |  |
|  | Julia Houlton |  |  |
|  | Louise Gallie |  |  |
|  | Mohammad Jasimuddin |  |  |
|  | Michele Bell |  |  |
|  | Alan Mansell |  |  |
|  | Olivia Wills |  |  |
|  | Evelyn Mcgow |  |  |
|  | Michael Selby |  |  |
|  | Michael Goldstone |  |  |
|  | Susan Stuart |  |  |
|  | Hannah Burden |  |  |
|  | Rachel Kerr |  |  |
|  | Kevin Thomas Stevens |  |  |
|  | Judith Martin |  |  |
|  | Samantha Drewett |  |  |
|  | Gordon Ellison |  |  |
| **North America** |  |  |  |
| **Canada** |  |  |  |
| *Healthcare Professionals* |  |  |  |
|  | Carole Thompson | Critical Care Dietitian |  |
|  | Melanie Hart | Clinical Dietitian |  |
|  | Melanie Vachon | Dietician |  |
|  | Kaitlin Szpytman | Clinical Dietitian |  |
|  | Kristen Maceachern | Dietician |  |
| **USA** |  |  |  |
| *Clinical Researchers* |  |  |  |
|  | Laura Niederer | Clinical Research Coordinator - Dietitian |  |
|  | Christopher Messenger | Acute Care Registered Dietitian | Assistant Professor |
|  | Vijay Srinivasan | Attending Physician | Associate Professor of Anesthesiology and Critical Care |
|  | Liam Mckeever | Dietician | Adjunct Professor of Nutrition |
|  | Todd Rice | Physician/Intensivist | Associate Professor of Medicine |
|  | Kenneth Christopher | Intensive Care Renal Clinician | Clinical Researcher / Educator |
|  | Charlene Compher | Dietitian Specialist | Professor Of Nutrition Science |
| *Healthcare Professionals* |  |  |  |
|  | Cindi Fiechtner | Registered Dietitian |  |
|  | Jeffrey Mechanick | Endocrinologist |  |
|  | Rachel Ludke | Clinical Dietitian |  |
|  | Michelle Curll | Registered Dietitian |  |
|  | Kelli Farr | Dietitian |  |
|  | Diana Stoermann | Dietitian |  |
|  | Stephanie Zamudio | Intensive Care Dietitian/Nutrition Support Clinician |  |
|  | April Church | Dietitian |  |
|  | Katy Argo | Registered Dietitian |  |
|  | Oluwakemi Adeola | Clinical Assistant Professor/Registered Dietitian |  |
|  | Jennifer Johanek | Clinical Dietitian |  |
|  | Jennifer Crain | Registered Dietitian |  |
|  | Erin Nella | Intensive Care Dietitian |  |
|  | Tori Nicolli | Clinical Dietitian |  |
|  | Brandee Grenda | Clinical Nutritionist |  |
|  | Katharine Parker |  |  |
|  | Mary Russell | Dietitian |  |
|  | Amy Quinn | Pharmacist |  |
|  | Dale Needham | Critical Care Physician |  |
|  | Martin Rosenthal | Trauma Surgeon and Critical Care |  |
|  | Gina Wilderspin | Dietitian |  |
|  | Mykel Moody | Dietitian |  |
|  | Lauren Clark | Critical Care Dietitian |  |
|  | Kathleen Gura | Pharmacist |  |
|  | Kim Sabino | Critical Care Dietitian |  |
|  | Jessica Engelbrecht | Critical Care Registered Dietitian |  |
|  | Gertrudis Baptista | Titular Professor of Medicine |  |
|  | Sara Hennessy | Physician |  |
|  | Kris Mogensen | Clinical Dietitian |  |
| *Patients or Caregivers* |  |  |  |
|  | Alice White |  |  |
| **South America** |  |  |  |
| **Brazil** |  |  |  |
| *Clinical Researchers* |  |  |  |
|  | Denise Philomene Joseph van Aanholt | Dietitian | Auditor In Nutritional Therapy |
|  | Thiago Goncalves | Doctor | Physician |
|  | Dan L. Waitzberg | Physician | Associate Professor |
|  | Maria Cristina Gonzalez | Physician | Professor |
|  |  |  |  |
|  |  |  |  |
| *Healthcare Professionals* |  |  |  |
|  | Ricardo Schilling Rosenfeld | Nutrition Support Medical Director/ Intensive Care Physician |  |
|  | Pedro Eder Portari Filho | Surgeon/Medicine |  |
|  | Nara Lucia Andrade Lopes | Nutritionist |  |
|  | Maria Isabel Toulson Davisson Correia | Professor Of Surgery |  |
|  | Juliana Tepedino Martins Alves | Medical Clinical Nutrition |  |
| **Mexico** |  |  |  |
| *Healthcare Professionals* |  |  |  |
|  | Adriana Martínez Terrazas | Doctor |  |

***Consensus Meeting participants***

**Supplementary Information Table S5 Consensus meeting participants**

| **Continent, Country, Stakeholder Group** | **Representative** |
| --- | --- |
| **Asia** |  |
| **Singapore** |  |
| *Clinical Researchers* |  |
|  | Andrew Li |
| **Malaysia** |  |
| *Clinical Researchers* |  |
|  | Mazuin Kamarul Zaman |
|  | Lee Zheng Yii |
| *Patients or Caregivers* |  |
|  | David K T Chua |
| **Philippines** |  |
| *Healthcare Professionals* |  |
|  | Gulbin Aygencel |
| **Europe** |  |
| **Belgium** |  |
| *Clinical Researchers* |  |
|  | Jean Charles Preiser |
|  | Jan Gunst |
|  | Anne-Françoise Rousseau |
|  | Elisabeth De Waele |
|  | Rik Gosselink |
|  | Michael Casaer |
| *Healthcare Professionals* |  |
|  | Karolien Dams |
|  | Vincent Fraipont |
|  | Xavier Wittebole |
| **Estonia** |  |
| *Clinical Researchers* |  |
|  | Prof Alastair Forbes |
| *Healthcare Professionals* |  |
|  | Kadri Tamme |
| **France** |  |
| *Healthcare Professionals* |  |
|  | Ronan Thibault |
| **Germany** |  |
| *Clinical Researchers* |  |
|  | Steffen Weber-Carstens |
|  | Stefan Schaller |
|  | Christian Stoppe |
| *Healthcare Professionals* |  |
|  | Aileen Hill |
| **Italy** |  |
| *Clinical Researchers* |  |
|  | Nicola Latronico |
| **Netherlands** |  |
| *Clinical Researchers* |  |
|  | Rob van Gassel |
| *Healthcare Professionals* |  |
|  | Arthur van Zanten |
| *Patients or Caregivers* |  |
|  | J. Valentijn |
|  | Marjolein Siebel |
| **Russia** |  |
| *Healthcare Professionals* |  |
|  | Mikhail Kirov |
| **Spain** |  |
| *Healthcare Professionals* |  |
|  | Clara Vaquerizo |
| **Switzerland** |  |
| *Clinical Researchers* |  |
|  | Annika Reintam Blaser |
| **United Kingdom** |  |
| *Clinical Researchers* |  |
|  | Zudin Puthucheary |
| *Healthcare Professionals* |  |
|  | Danielle Bear |
|  | Robert Wise |
|  | Emma Gaskin |
| *Patients or Caregivers* |  |
|  | Mark Hudson |
|  | Francesco Palma |
|  | Rebecca Langley |
|  | Mohammad Jasimuddin |
|  | Kevin Thomas Stevens |
|  | Judith Martin |
|  | Samantha Drewett |
| **North America** |  |
| Canada |  |
| *Healthcare Professionals* |  |
|  | Carole Thompson |
|  | Kristen Maceachern |
| **USA** |  |
| *Clinical Researchers* |  |
|  | Christopher Messenger |
|  | Todd Rice |
|  | Charlene Compher |
|  | Beth Taylor |
| *Healthcare Professionals* |  |
|  | Michelle Curll |
|  | Oluwakemi Adeola |
|  | Dale Needham |
|  | Beth Taylor |
| ***South America*** |  |
| ***Brazil*** |  |
| *Clinical Researchers* |  |
|  | Denise Philomene Joseph van Aanholt |
|  | Maria Cristina Gonzalez |
| *Healthcare Professionals* |  |
|  | Ricardo Schilling Rosenfeld |

***Results***

***Systematic Review***

*Characteristics of included randomised controlled trials*

All 25 studies were parallel group RCTs, recruiting medical (12%), surgical (4%), mixed (68%), neurosurgical (12%), or liver (4%) critically ill patients. Only 14 (56%) were blinded and 9 (36%) multi-centred.

*Summary table*

Supplementary Information Table S6 Characteristics of trials included in systematic review

| Author, Year | Region | Recruitment | Study population | Number of participants | Main study topic | Duration of intervention (days) |
| --- | --- | --- | --- | --- | --- | --- |
| Chapman et al., 2018 [2] | Oceania | Multicentre | Mixed ICU | 3951 | Composition of nutritional strategy | 6 |
| Fetterplace et al., 2018 [3] | Oceania | Single-centre | Mixed ICU | 60 | Clinical nutrition strategy | 8 |
| Danielis et al., 2019 [4] | Europe | Single-centre | Mixed ICU | 40 | Composition of nutritional strategy | 7 |
| Nakamura et al., 2019 [5] | Asia | Single-centre | Mixed ICU | 88 | Nutritional supplementation | 10 |
| Lu et al., 2020 [6] | Asia | Single-centre | Neurosurgical ICU | 28 | Clinical nutrition strategy | 3 |
| Donoghue et al., 2019 [7] | Africa | Single-centre | Mixed ICU | 75 | Composition of nutritional strategy | 5 |
| Hejazi et al., 2018 [8] | Asia | Single-centre | Mixed ICU | 80 | Nutritional supplementation | 10 |
| Wandrag et al., 2019 [9] | Europe | Single-centre | Mixed ICU | 8 | Nutritional supplementation | 14 |
| Mousavian et al., 2020 [10] | Asia | Single-centre | Neurosurgical ICU | 68 | Clinical nutrition strategy | 14 |
| Wang et al., 2020 [11] | Asia | Single-centre | Medical ICU | 150 | Clinical nutrition strategy | 6 |
| Fazilatyet al., 2018 [12] | Asia | Single-centre | Mixed ICU | 40 | Nutritional supplementation | 10 |
| Tihista et al. 2018 [13] | Europe | Single-centre | Medical ICU | 106 | Composition of nutritional strategy | 14 |
| de Azevedo et al., 2019 [14] | South America | Single-centre | Mixed ICU | 138 | Clinical nutrition strategy | 10 |
| Yeh et al., 2019 [15] | America | Multicentre | Surgical ICU | 36 | Clinical nutrition strategy | 14 |
| Ridley et al., 2018 [16] | Oceania | Multicentre | Mixed ICU | 100 | Clinical nutrition strategy | 7 |
| van Zanten et al., 2018 [17] | Europe | Multicentre | Mixed ICU | 44 | Composition of nutritional strategy | 10 |
| Gonzalez-Granda et al., 2018 [18] | Europe | Single-centre | Medical ICU | 76 | Clinical nutrition strategy | 18 |
| Zhu et al., 2018 [19] | Asia | Single-centre | Mixed ICU | 141 | Clinical nutrition strategy | 7 |
| McNelly et al., 2020 [20] | Europe | Multicentre | Mixed ICU | 127 | Clinical nutrition strategy | 10 |
| Schmidt et al., 2018 [21] | Europe | Multicentre | Neuro ICU | 119 | Composition of nutritional strategy | 30 |
| Yaseen M Aarabi  (ClinicalTrials.gov) | Asia | Multicentre | Mixed ICU | - | Nutritional supplementation | ICU stay |
| Jaya Joy Benjamin  (ClinicalTrials.gov) | Asia | Single-centre | Liver ICU | - | Clinical nutrition strategy | ICU stay |
| Daren K. Heyland  (ClinicalTrials.gov) | Oceania | Multicentre | Mixed ICU | - | Composition of nutritional strategy | 28 |
| Marcel CG van de Poll  (ClinicalTrials.gov) | Europe | Multicentre | Mixed ICU | - | Composition of nutritional strategy | ICU stay |
| Mette M Berger  (ClinicalTrials.gov) | Europe | Single-centre | Mixed ICU | - | Nutritional supplementation | 30 |

ICU = intensive care unit

*Interventions tested*

**Supplementary Information Table S7 Interventions tested in trials identified in the systematic review**

| Intervention | N (%) of 25 RCTs |
| --- | --- |
| Nutrition strategy  Optimization (EN)  Prescription (EN)  Optimization (PN)  PN versus Standard care  Intermittent feeding  Post-pyloric feeding | 11(44)  1  4  2  1  2  1 |
| Nutritional Composition  EN  PN  Mixed | 8(32%)  6  1  1 |
| Nutritional Supplementation  Glutamine  Lipoic Acid  Β-hydroxy-β-methylbutyric acid  Protein  Leucine  β-glucan | 6 (24%)  1  1  1  1  1  1 |

EN = enteral nutrition; PN = parenteral nutrition

*Risk of Bias*

Risk of bias was assessed using the Cochrane risk-of-bias tool for RCTs (Rob-2) [22]. Summary data are shown in SI Figure S6 and the domains included in SI Figure S3. Only three studies had a low risk of bias. The domain “*Blinding of outcome assessment*” was scored as a high risk of bias in 55% (11/20) of trials.

Supplementary Information Fig S2 Summary of risk of bias assessment


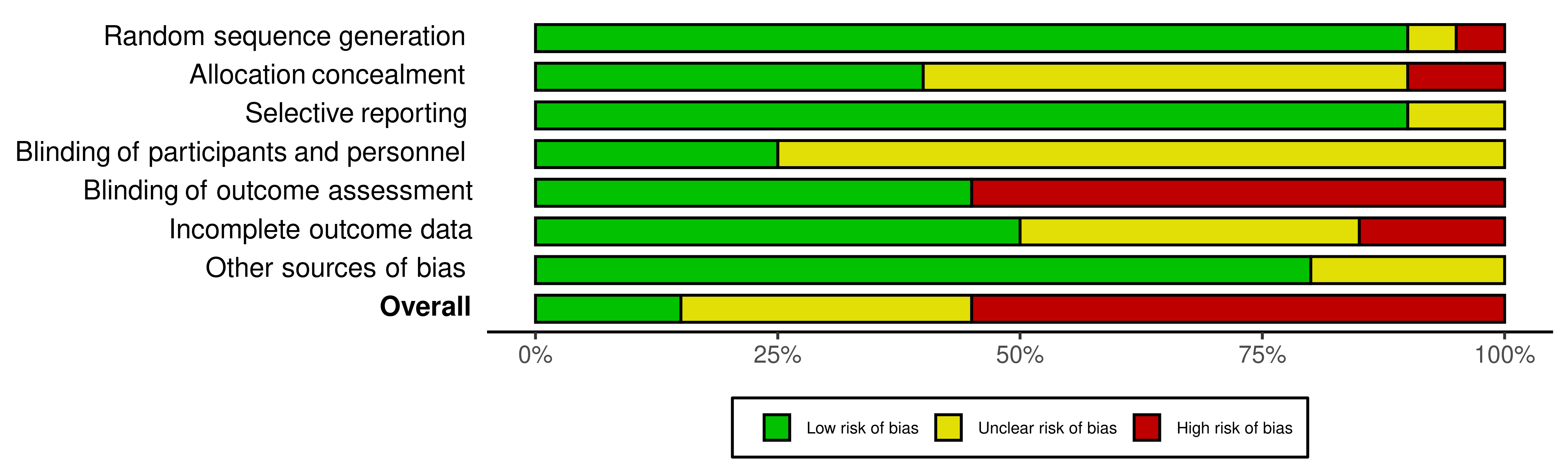


Supplementary Information Fig S3 Summary of domains assessed for risk of bias per study

*
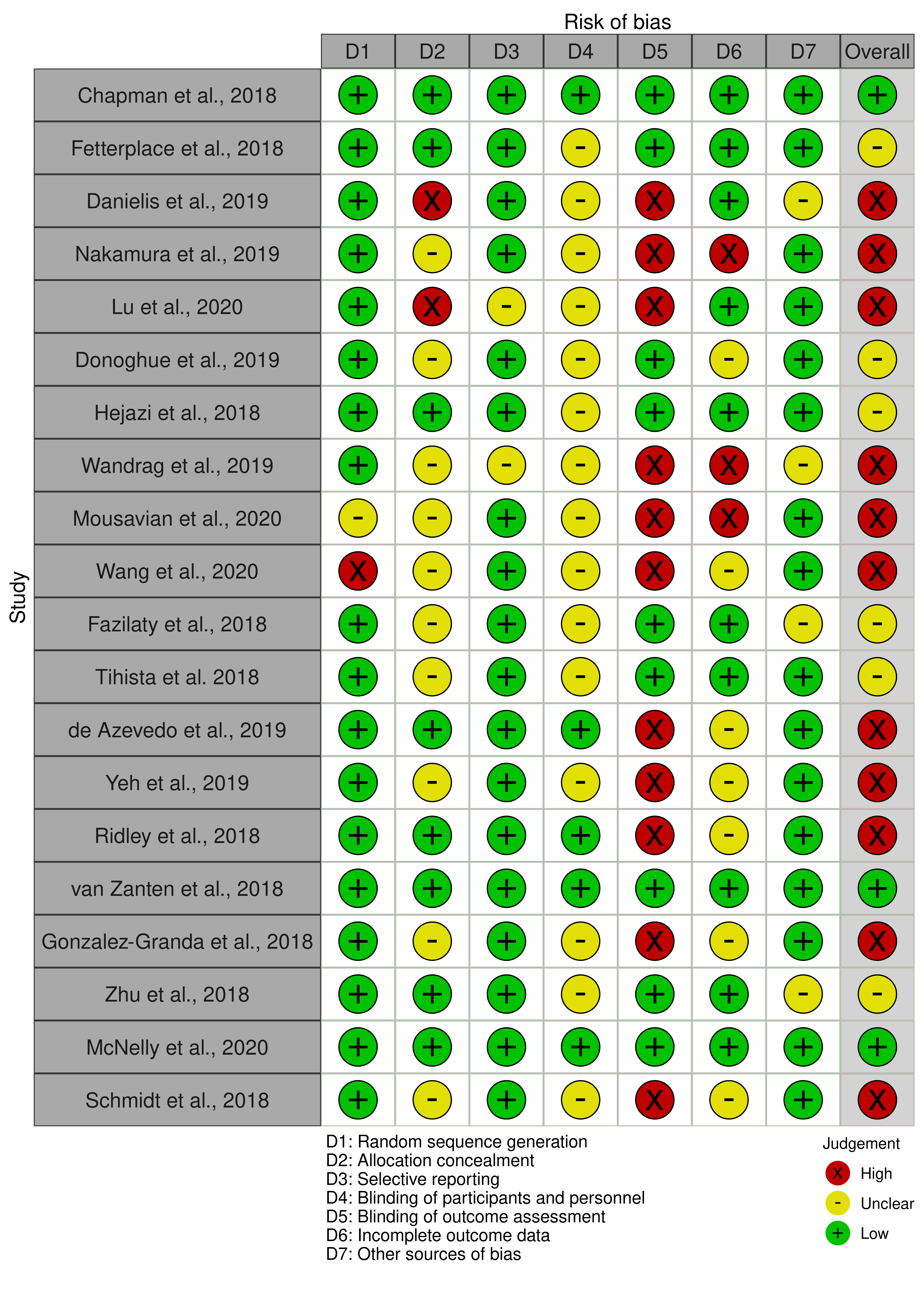
*

*Limitations of Systematic Review*

The review was an update of a previous review and therefore included a small number of studies over a relatively short period of time. This may have contributed to the heterogeneity seen in our results. Many of the studies were poorly conducted or poorly reported but our overall goal was to obtain an up-to-date overview of the outcomes used in RCTs, regardless of their quality or results.

***Results***

***Delphi Process***

*Number of participants*

**Supplementary Information Figure S4 Number of participants in each stage**

**
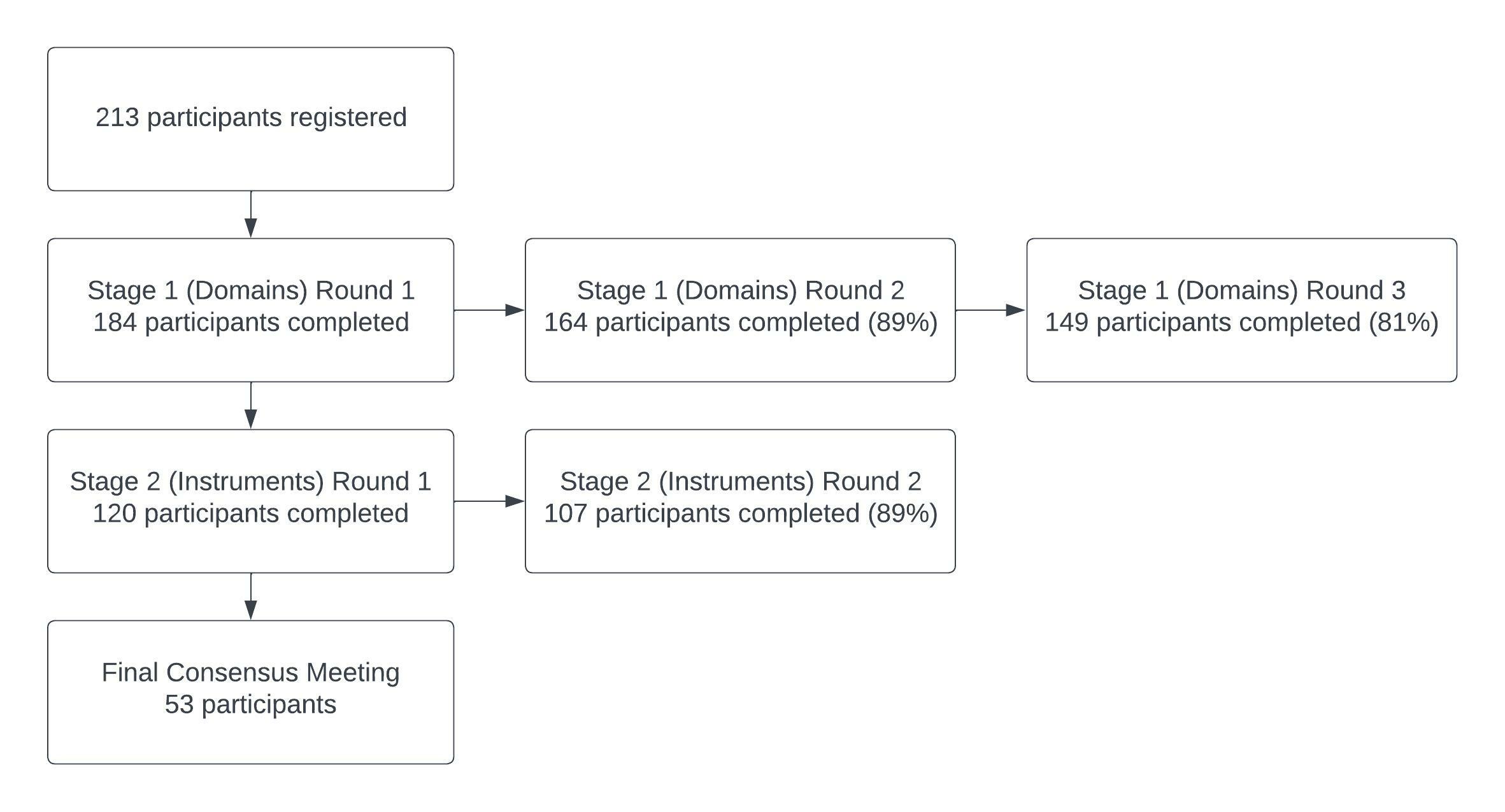
**

(%) = percentage of participants retained from first round of each stage

***Core Outcome Set - Domains***

Domains considered but were excluded from the Delphi Stage 1 by the steering committee:

1.Mental health conditions and symptoms

2. Pain

3. Bone health

4. Sleep quality

5. Endocrine and metabolic disorders

6. Skin scars

7. Relational and sexual functioning

8. Eating habits

9. Cardiovascular function and symptoms (considered part of organ dysfunction and physical function)

10. Respiratory function and symptoms (considered part of organ dysfunction and physical function

*Final domain results for 30 days post randomisation*

**Supplementary Information Table S8 Final results of domain performance at 30 day post randomisation**

| Domain | Score^#^ | Proportion (%) of stakeholders rating the domain as≥7 on a 9-point Likert Scale | | | |
| --- | --- | --- | --- | --- | --- |
|  |  | All (n=164) | Healthcare Professionals (n=90) | Clinical Researchers (n=41) | Patients and Caregivers (n=33) |
| Survival | 8.09 (1.30) | 89 | 92 | 83 | 88 |
| Physical function and symptoms | 7.64 (1.24) | 85 | 83 | 83 | 91 |
| Infection | 7.54 (1.38) | 80 | 85 | 65 | 82 |
| Organ dysfunction | 7.46 (1.65) | 79 | 81 | 73 | 82 |
| Muscle and/or nerve function | 7.45 (1.46) | 79 | 80 | 75 | 79 |
| Nutritional status* | 7.42 (1.46) | 79 | 89 | 62 | 72 |
| Wound healing | 7.28 (1.60) | 73 | 82 | 58 | 67 |
| Frailty | 6.84 (1.79) | 68 | 70 | 60 | 76 |
| GI function and symptoms | 6.95 (1.45) | 66 | 72 | 50 | 70 |
| Body composition | 6.96 (1.49) | 64 | 70 | 63 | 52 |
| Swallowing* | 6.53 (1.57) | 61 | 65 | 43 | 73 |
| Activities of daily living | 6.62 (1.63) | 57 | 55 | 63 | 55 |
| Cognitive function and symptoms | 6.55 (1.54) | 55 | 47 | 53 | 76 |
| Inflammation* | 6.30 (1.55) | 52 | 54 | 32 | 70 |
| Fatigue | 6.35 (1.59) | 49 | 46 | 45 | 64 |
| Discharge destination | 6.47 (1.65) | 48 | 47 | 58 | 39 |
| Health care utilisation | 6.35 (1.69) | 46 | 43 | 50 | 48 |
| Mental health | 6.06 (1.73) | 44 | 43 | 30 | 64 |
| Satisfaction with life | 5.96 (1.70) | 34 | 31 | 35 | 39 |
| Return to employment | 5.56 (2.00) | 34 | 37 | 40 | 21 |
| Microbiome | 5.60 (1.97) | 29 | 28 | 20 | 39 |
| Family opinion | 5.35 (1.86) | 29 | 26 | 33 | 33 |
| Physical vitality | 5.19 (1.85) | 26 | 29 | 10 | 33 |
| Bone health | 5.25 (1.75) | 23 | 20 | 15 | 39 |
| Sexual health | 3.87 (1.86) | 7 | 4 | 10 | 9 |

^#^Mean score (standard deviation). *domains that required a third round to ensure two rounds of voting (n=149)

*Final domain results for 90 days post randomisation*

**Supplementary Information Table S9 Final results of domain performance at 90 day post randomisation**

| Domain | Score^#^ | Proportion (%) of stakeholders rating the domain as≥7 on a 9-point Likert Scale | | | |
| --- | --- | --- | --- | --- | --- |
|  |  | All (n=164) | Healthcare Professionals (n=90) | Clinical Researchers (n=41) | Patients and Caregivers (n=33) |
| Physical function and symptoms | 7.99 (1.13) | 89 | 92 | 85 | 85 |
| Survival | 8.09 (1.42) | 85 | 84 | 85 | 88 |
| Activities of daily living | 7.76 (1.33) | 83 | 88 | 83 | 70 |
| Nutritional status* | 7.48 (1.43) | 83 | 89 | 73 | 79 |
| Muscle and/or nerve function | 7.65 (1.30) | 82 | 82 | 78 | 88 |
| Frailty | 7.48 (1.50) | 79 | 84 | 70 | 73 |
| Body composition | 7.24 (1.42) | 74 | 84 | 63 | 61 |
| Organ dysfunction | 7.16 (1.86) | 74 | 74 | 65 | 82 |
| Cognitive function and symptoms | 7.05 (1.64) | 66 | 58 | 65 | 85 |
| Wound healing | 6.89 (1.79) | 65 | 78 | 40 | 58 |
| Satisfaction with life | 6.99 (1.65) | 63 | 60 | 70 | 61 |
| Swallowing | 6.65 (1.75) | 63 | 65 | 53 | 67 |
| Fatigue | 6.80 (1.59) | 61 | 56 | 60 | 76 |
| GI function and symptoms | 6.65 (1.70) | 60 | 64 | 48 | 64 |
| Discharge destination | 6.86 (1.69) | 57 | 56 | 70 | 45 |
| Infection | 6.69 (1.69) | 55 | 51 | 55 | 67 |
| Health care utilisation | 6.62 (1.63) | 55 | 60 | 58 | 39 |
| Return to employment | 6.62 (1.58) | 54 | 57 | 60 | 33 |
| Mental health | 6.31 (1.77) | 49 | 48 | 38 | 64 |
| Inflammation* | 6.06 (1.62) | 48 | 47 | 27 | 77 |
| Microbiome | 5.58 (2.03) | 32 | 30 | 23 | 42 |
| Physical vitality | 5.48 (1.85) | 30 | 33 | 15 | 39 |
| Bone health | 5.71 (1.67) | 27 | 21 | 20 | 48 |
| Family opinion | 5.28 (1.79) | 26 | 26 | 23 | 30 |
| Sexual health | 4.26 (2.08) | 15 | 11 | 15 | 21 |

^#^Mean score (standard deviation). *domains that required a third round to ensure two rounds of voting (n=149)

*Variation in scores between groups of participants for outcome domains at 30 days post randomisation*

**Supplementary Information Table S10 Mean (standard deviation) of scores per stakeholder group for outcome domains at 30 days post randomisation**

| Domains at 30 days post randomisation | Healthcare Professionals | Clinical Researchers | Patients and Caregivers | Mean |
| --- | --- | --- | --- | --- |
| Survival | 8.13 (1.2) | 7.93 (1.5) | 8.22 (1.3) | 8.09 (1.30) |
| Physical function and symptoms | 7.57 (1.2) | 7.73 (1.4) | 7.73 (1.1) | 7.64 (1.24) |
| Infection | 7.62 (1.3) | 7.22 (1.6) | 7.85 (1.3) | 7.54 (1.38) |
| Organ dysfunction | 7.54 (1.4) | 7.12 (2.2) | 7.81 (1.6) | 7.46 (1.65) |
| Muscle and/or nerve function | 7.51 (1.3) | 7.22 (1.7) | 7.61 (1.6) | 7.45 (1.46) |
| Nutritional status* | 7.68 (1.4) | 6.43 (2.1) | 7.33 (1.5) | 7.42 (1.46) |
| Wound healing | 7.49 (1.4) | 6.68 (2.0) | 7.42 (1.4) | 7.28 (1.60) |

*denotes significant differences between clinical researchers and patients and caregivers

*Variation in scores between groups of participants for outcome domains at 90 days post randomisation*

**Supplementary Information Table S11 Mean (standard deviation) of scores per stakeholder group for outcome domains at 90 days post randomisation**

| Domains at 90 days post randomisation | Healthcare Professionals | Clinical Researchers | Patients and Caregivers | Mean |
| --- | --- | --- | --- | --- |
| Survival | 8.11 (1.3) | 8.05 (1.7) | 8.09 (1.3) | 8.09 (1.42) |
| Physical function and symptoms | 8.04 (1.0) | 8.05 (1.4) | 7.76 (1.2) | 7.99 (1.13) |
| Activities of daily living^#^ | 7.84 (1.1) | 7.8 (1.7) | 7.33 (1.4) | 7.76 (1.33) |
| Muscle and/or nerve function | 7.69 (1.3) | 7.39 (1.5) | 7.91 (1.1) | 7.65 (1.30) |
| Nutritional status | 7.66 (1.4) | 6.68 (2.1) | 7.31 (1.4) | 7.48 (1.43) |
| Frailty | 7.62 (1.3) | 7.32 (1.9) | 7.27 (1.5) | 7.48 (1.50) |
| Body composition^#^ | 7.59 (1.2) | 6.8 (1.7) | 6.76 (1.3) | 7.24 (1.42) |
| Organ dysfunction^#$^ | 7.24 (1.7) | 6.63 (2.4) | 7.81 (1.2) | 7.16 (1.86) |

^#^denotes significant differences between health care professionals and patients and caregivers; ^$^denotes significant differences between patients and caregivers and both health care professionals and clinical researchers

***Core Outcome Set - Measurement Instruments***

*Delphi measurement instrument results pre-consensus meeting for 30 days post randomisation*

**Supplementary Information Table S12 Final results of measurement instrument performance at 30 day post randomisation**

| Domain | Measurement Instrument | Score^#^ | Proportion (%) of stakeholders rating the domain as ≥7 on a 9-point Likert Scale | | | |
| --- | --- | --- | --- | --- | --- | --- |
|  |  |  | All  (n=107) | Healthcare Professionals (n=57) | Clinical Researchers (n=28) | Patients and Caregivers (n=24) |
| Physical function and symptoms | Physical component score of the 36-Item short form survey | 7.23 (1.41) | 75 | 75 | 79 | 71 |
|  | 30 second sit to stand | 6.77 (1.61) | 65 | 73 | 68 | 46 |
|  | 6-minute walk test | 6.67 (1.75) | 62 | 75 | 46 | 50 |
|  | 2-minute walk test | 6.41 (1.82) | 55 | 56 | 64 | 42 |
|  | Short physical performance battery | 6.68 (1.67) | 52 | 58 | 43 | 50 |
|  | 4-minute walk test | 6.20 (1.88) | 46 | 45 | 43 | 50 |
| Infection | Administration of antibiotics | 6.96 (1.54) | 69 | 73 | 54 | 79 |
|  | Sepsis 3.0 definition | 7.06 (1.78) | 67 | 60 | 64 | 88 |
|  | Positive blood cultures | 6.54 (1.76) | 52 | 49 | 50 | 63 |
|  | ECDC definition | 6.61 (1.70) | 46 | 38 | 39 | 71 |

^#^Mean score (standard deviation). ECDC = European centre for disease prevention and control

*Variation in scores between groups of participants for measurement instruments at 30 days post randomisation*

**Supplementary Information Table S13 Mean (standard deviation) of scores per stakeholder group for measurement instruments at 30 days post randomisation**

| Measurement Instruments at 30 days post randomisation | Healthcare Professionals | Clinical Researchers | Patients and Caregivers | Mean |
| --- | --- | --- | --- | --- |
| Physical component score of the 36-item short form survey | 7.42 (1.29) | 7.11 (1.81) | 7.29 (1.43) | 7.23 (1.41) |
| Sepsis 3.0 definition^*#^ | 7.09 (1.78) | 6.64 (2.20) | 8.21 (1.18) | 7.06 (1.78) |
| Administration of antibiotics^*$^ | 7.16 (1.40) | 6.21 (1.93) | 7.63 (1.17) | 6.96 (1.54) |
| 30 second sit to stand | 6.98 (1.41) | 6.71 (2.02) | 6.33 (1.49) | 6.77 (1.61) |
| 6-minute walk test^$^ | 7.07 (1.62) | 6.07 (2.18) | 6.58 (1.38) | 6.67 (1.75) |

*denotes significant differences between clinical researchers and patients and caregivers; ^#^denotes significant differences between health care professionals and patients and caregivers; ^$^denotes significant differences between clinical researchers and health care professionals

*Delphi measurement instrument results pre-consensus meeting for 90 days post randomisation*

**Supplementary Information Table S14 Final results of measurement instrument performance at 90 day post randomisation**

| Domain | Measurement Instrument | Score^#^ | Proportion (%) of stakeholders rating the domain as≥7 on a 9-point Likert Scale | | | |
| --- | --- | --- | --- | --- | --- | --- |
|  |  |  | All  (n=107) | Healthcare Professionals (n=57) | Clinical Researchers (n=28) | Patients and Caregivers (n=24) |
| Physical function and symptoms | Physical component score of the 36-Item short form survey | 7.45 (1.22) | 80 | 84 | 71 | 83 |
|  | 6-minute walk test | 7.16 (1.51) | 72 | 84 | 54 | 67 |
|  | 30 second sit to stand | 6.87 (1.51) | 71 | 80 | 61 | 63 |
|  | Short physical performance battery | 6.94 (1.66) | 66 | 67 | 54 | 79 |
|  | 2-minute walk test | 6.54 (1.80) | 65 | 62 | 64 | 75 |
|  | 4-minute walk test | 6.42 (1.84) | 55 | 64 | 39 | 54 |
| Muscle and/or nerve function | Handgrip strength | 6.92 (1.49) | 64 | 78 | 57 | 38 |
|  | Medical research council sum score | 6.88 (1.44) | 56 | 51 | 54 | 71 |
|  | Quadriceps force | 6.33 (1.59) | 48 | 60 | 36 | 33 |
|  | Biceps dynamometry | 5.77 (1.74) | 36 | 40 | 29 | 33 |
|  | Electromyography / nerve conduction studies | 5.26 (1.76) | 16 | 15 | 11 | 25 |
| Activities of daily living | Barthel index | 7.26 (1.36) | 78 | 75 | 75 | 88 |
|  | ADL/IADL combination score | 7.24 (1.42) | 74 | 73 | 75 | 75 |
|  | Katz index | 6.92 (1.49) | 63 | 58 | 71 | 63 |
| Nutritional Status | GLIM criteria | 7.42 (1.55) | 76 | 80 | 64 | 79 |
|  | Body mass index | 6.59 (1.73) | 57 | 58 | 50 | 63 |
|  | Subjective global assessment | 6.68 (1.69) | 52 | 49 | 46 | 67 |
|  | Bioelectrical impedance analysis | 5.96 (1.82) | 40 | 42 | 39 | 38 |

^#^Mean score (standard deviation). ECDC = European centre for disease prevention and control. ADL = Activities of daily living; IADL = Instrumental activities of daily living; GLIM = Global leadership initiative on malnutrition

*Variation in scores between groups of participants for measurement instruments at 90 days post randomisation*

**Supplementary Information Table S15 Mean (standard deviation) of scores per stakeholder group for measurement instruments at 90 days post randomisation**

| Measurement Instruments at 90 days post randomisation | Healthcare Professionals | Clinical Researchers | Patients and caregivers | Mean |
| --- | --- | --- | --- | --- |
| Physical component score of the 36-Item short form survey | 7.63 (1.09) | 7.18 (1.63) | 7.36 (0.85) | 7.45 (1.22) |
| GLIM criteria^$^ | 7.68 (1.27) | 6.74 (2.10) | 7.64 (1.14) | 7.42 (1.55) |
| Barthel index | 7.35 (1.30) | 7.00 (1.74) | 7.39 (0.89) | 7.26 (1.36) |
| ADL/IADL combination score | 7.21 (1.45) | 7.22 (1.63 | 7.32 (1.13) | 7.24 (1.42) |
| 30 second sit to stand^$^ | 7.15 (1.24) | 6.32 (1.96) | 6.88 (1.33) | 7.16 (1.51) |
| Short physical performance battery^*^ | 7.06 (1.55) | 6.26 (2.09) | 7.50 (0.96) | 6.94 (1.66) |
| Handgrip strength^#^ | 7.27 (1.37) | 6.61 (1.85) | 6.43 (1.08) | 6.92 (1.49) |
| Katz index | 7.00 (1.40) | 6.89 (1.79) | 6.77 (1.27) | 6.92 (1.49) |
| 6-minute walk test^$^ | 7.54 (1.24) | 6.46 (2.03) | 7.13 (1.08) | 6.87 (1.51) |

*denotes significant differences between clinical researchers and patients and caregivers; ^#^denotes significant differences between health care professionals and patients and caregivers; ^$^denotes significant differences between clinical researchers and health care professionals. ADL = Activities of daily living; IADL = Instrumental activities of daily living; GLIM = Global leadership initiative on malnutrition

***References***

1. Taverny G, Lescot T, Pardo E, Thonon F, Maarouf M, Alberti C. Outcomes used in randomised controlled trials of nutrition in the critically ill: a systematic review. Crit Care Lond Engl. 2019;23:12.

2. Chapman M, Peake SL, Bellomo R, Davies A, Deane A, Horowitz M, et al. Energy-Dense versus Routine Enteral Nutrition in the Critically Ill. N Engl J Med. United States; 2018;379:1823–34.

3. Fetterplace K, Deane AM, Tierney A, Beach LJ, Knight LD, Presneill J, et al. Targeted Full Energy and Protein Delivery in Critically Ill Patients: A Pilot Randomized Controlled Trial (FEED Trial). JPEN J Parenter Enteral Nutr. United States; 2018;42:1252–62.

4. Danielis M, Lorenzoni G, Azzolina D, Iacobucci A, Trombini O, De Monte A, et al. Effect of Protein-Fortified Diet on Nitrogen Balance in Critically Ill Patients: results from the OPINiB Trial. Nutrients [Internet]. 2019;11. Available from: https://www.cochranelibrary.com/central/doi/10.1002/central/CN-01937713/full

5. Nakamura K, Kihata A, Naraba H, Kanda N, Takahashi Y, Sonoo T, et al. β-Hydroxy-β-methylbutyrate, Arginine, and Glutamine Complex on Muscle Volume Loss in Critically Ill Patients: a Randomized Control Trial. JPEN J Parenter Enteral Nutr. 2020;44:205‐212.

6. Lu K, Zeng F, Li Y, Chen C, Huang M. A more physiological feeding process in ICU: intermittent infusion with semi-solid nutrients (CONSORT-compliant). Medicine (Baltimore). 2018;97:e12173.

7. Donoghue V, Schleicher G, Spruyt M, Malan L, Nel D, Calder P, et al. Four-oil intravenous lipid emulsion effect on plasma fatty acid composition, inflammatory markers and clinical outcomes in acutely ill patients: a randomised control trial (Foil fact). Clin Nutr Edinb Scotl. 2019;38:2583‐2591.

8. Hejazi N, Mazloom Z, Zand F, Rezaianzadeh A, Nikandish R. The Beneficial Effects of α-Lipoic Acid in Critically Ill Patients: a Prospective, Randomized, Double-Blind, Placebo-Controlled Trial. Asian J Anesthesiol. 2018;56:45‐55.

9. Wandrag L, Brett S, Frost G, To M, Loubo E, Jackson N, et al. Leucine-enriched essential amino acid supplementation in mechanically ventilated trauma patients: a feasibility study. Trials. 2019;20:561.

10. Mousavian S, Pasdar Y, Ranjbar G, Jandari S, Akhlaghi S, Almasi A, et al. Randomized Controlled Trial of Comparative Hypocaloric vs Full-Energy Enteral Feeding During the First Week of Hospitalization in Neurosurgical Patients at the Intensive Care Unit. JPEN J Parenter Enteral Nutr. 2020;44:1475‐1483.

11. Wang C, Fu P, Chao W, Wang W, Chen C, Huang Y. Full Versus Trophic Feeds in Critically Ill Adults with High and Low Nutritional Risk Scores: a Randomized Controlled Trial. Nutrients [Internet]. 2020;12. Available from: https://www.cochranelibrary.com/central/doi/10.1002/central/CN-02202031/full

12. Fazilaty Z, Chenari H, Shariatpanahi Z. Effect of ß-glucan on serum levels of IL-12, hs-CRP, and clinical outcomes in multiple-trauma patients: a prospective randomized study. Ulus Travma Ve Acil Cerrahi Derg Turk J Trauma Emerg Surg TJTES. 2018;24:287‐293.

13. Tihista S, Echavarría E. Effect of omega 3 polyunsaturated fatty acids derived from fish oil in major burn patients: a prospective randomized controlled pilot trial. Clin Nutr Edinb Scotl. 2018;37:107‐112.

14. Azevedo J, Lima H, Montenegro W, Souza S, Nogueira I, Silva M, et al. Optimized calorie and high protein intake versus recommended caloric-protein intake in critically ill patients: a prospective, randomized, controlled phase II clinical trial. Rev Bras Ter Intensiva. 2019;31:171‐179.

15. Yeh D, Ortiz L, Lee J, Chan J, McKenzie K, Young B, et al. PEP uP (Enhanced Protein-Energy Provision via the Enteral Route Feeding Protocol) in Surgical Patients-A Multicenter Pilot Randomized Controlled Trial. JPEN J Parenter Enteral Nutr. 2020;44:197‐204.

16. Ridley E, Davies A, Parke R, Bailey M, McArthur C, Gillanders L, et al. Supplemental parenteral nutrition versus usual care in critically ill adults: a pilot randomized controlled study. Crit Care Lond Engl. 2018;22:12.

17. van Zanten A, Petit L, De Waele J, Kieft H, de Wilde J, van Horssen P, et al. Very high intact-protein formula successfully provides protein intake according to nutritional recommendations in overweight critically ill patients: a double-blind randomized trial. Crit Care Lond Engl. 2018;22:156.

18. Gonzalez-Granda A, Schollenberger A, Haap M, Riessen R, Bischoff S. Optimization of Nutrition Therapy with the Use of Calorimetry to Determine and Control Energy Needs in Mechanically Ventilated Critically Ill Patients: the ONCA Study, a Randomized, Prospective Pilot Study. JPEN J Parenter Enteral Nutr. 2019;43:481‐489.

19. Zhu Y, Yin H, Zhang R, Ye X, Wei J. Gastric versus postpyloric enteral nutrition in elderly patients (age ≥ 75 years) on mechanical ventilation: a single-center randomized trial. Crit Care Lond Engl. 2018;22:170.

20. McNelly AS, Bear DE, Connolly BA, Arbane G, Allum L, Tarbhai A, et al. Effect of Intermittent or Continuous Feed on Muscle Wasting in Critical Illness: A Phase 2 Clinical Trial. Chest. United States; 2020;158:183–94.

21. Schmidt S, Kulig W, Winter R, Vasold A, Knoll A, Rollnik J. The effect of a natural food based tube feeding in minimizing diarrhea in critically ill neurological patients. Clin Nutr Edinb Scotl. 2019;38:332‐340.

22. Sterne JAC, Savović J, Page MJ, Elbers RG, Blencowe NS, Boutron I, et al. RoB 2: a revised tool for assessing risk of bias in randomised trials. BMJ. England; 2019;366:l4898.
